# Senescent BMSC‐Derived Thbs1 Drives Inflammaging and Impairs Bone Regeneration by Suppressing PINK1/Parkin‐Mediated Mitophagy in Macrophages

**DOI:** 10.1111/acel.70575

**Published:** 2026-06-08

**Authors:** Yifeng Xing, Jingjing Su, Yanjun Lin, Nengwen Huang, Sihui Zhang, Yuwei Zhou, Jie Lu, Weiping Chen, Kaixun He, Wenxiu Yuan, Yang Li, Geyuan Zheng, Pengyuan Hu, Dong Wu, Yanjing Ou, Jiang Chen

**Affiliations:** ^1^ Clinical Research Center for Oral Tissue Deficiency Diseases of Fujian Province, Fujian Key Laboratory of Oral Diseases, School and Hospital of Stomatology Fujian Medical University Fuzhou China; ^2^ Stomatological Key Laboratory of Fujian College and University and Research Center of Dental and Craniofacial Implants, School and Hospital of Stomatology Fujian Medical University Fuzhou China; ^3^ Stomatological Hospital of Xiamen Medical College Xiamen China; ^4^ Xiamen Key Laboratory of Stomatological Disease Diagnosis and Treatment Xiamen China; ^5^ Oral and Maxillofacial Surgery Fuzhou University Affiliated Provincial Hospital Fuzhou City China; ^6^ Department of Stomatology, Henan Provincial People's Hospital People's Hospital of Zhengzhou University Zhengzhou China

**Keywords:** bone regeneration, cellular senescence, inflammaging, macrophage polarization, mitophagy, thrombospondin‐1

## Abstract

The aging bone marrow microenvironment is characterized by chronic low‐grade inflammation (“inflammaging”), which disrupts skeletal homeostasis and impairs bone regeneration. However, the stromal–immune crosstalk mechanisms sustaining this pathological state remain poorly defined. Here, transcriptomic analysis identified thrombospondin‐1 (Thbs1) as a key upregulated component of the senescence‐associated secretory phenotype (SASP) in aged bone mesenchymal stromal cells (BMSCs). We demonstrate that BMSC‐derived Thbs1 drives pro‐inflammatory M1 macrophage polarization by suppressing PINK1/Parkin‐mediated mitophagy. Mechanistically, Thbs1 binds to the TGF‐β type II receptor (Tgfbr2) on macrophages to activate Smad3 signaling, which transcriptionally represses the mitophagy regulator *Pink1*. This repression leads to mitochondrial superoxide accumulation and redox imbalance, thereby skewing macrophages toward an M1‐like phenotype. These Thbs1‐activated M1 macrophages, in turn, secrete IL‐6, which activates the JAK/STAT3 pathway in BMSCs to inhibit osteogenic differentiation. Crucially, activated Stat3 directly binds the *Thbs1* promoter, establishing a self‐amplifying loop that perpetuates inflammaging and osteogenic decline. In vivo, AAV9‐mediated Thbs1 knockdown in aged rat bone defects restored mitochondrial homeostasis, promoted an M2 macrophage transition, and significantly enhanced bone repair. Our study reveals a vicious cycle involving the Thbs1/TGF‐β/Smad3/PINK1–IL‐6/JAK/STAT3 axis that sustains inflammaging and osteogenic decline, highlighting Thbs1 as a promising therapeutic target for age‐related bone regeneration.

## Graphical abstract

Aging BMSC‐derived Thbs1 activates the macrophage TGF‐β/Smad3 pathway to transcriptionally repress *Pink1*, impairing mitophagy and causing mitochondrial ROS accumulation. This redox imbalance drives M1 polarization and IL‐6 secretion. IL‐6 feeds back to BMSCs via the JAK/STAT3 pathway to inhibit osteogenesis and upregulate *Thbs1*, establishing a self‐amplifying loop that sustains inflammaging.

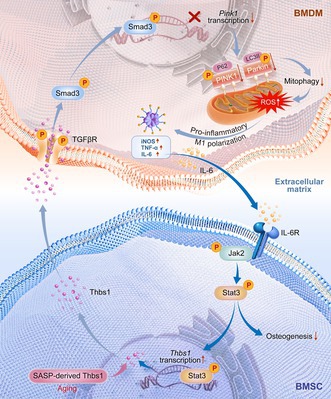

## 1 Introduction

As the global population ages rapidly, age‐related bone loss and microarchitectural deterioration have emerged as critical public health challenges (Guo et al. 2021). Senile osteoporosis and associated fractures not only increase morbidity and mortality in older adults but also impose a substantial economic burden on healthcare systems (Yu and Wang 2016; Harvey et al. 2010). Beyond the reduction in bone density and strength, a progressive decline in bone‐repair capacity is a defining feature of skeletal aging. This decline is fundamentally driven by inflammaging within the bone marrow microenvironment (BMM)—a hallmark of aging‐associated pathologies that acts as a major barrier to effective bone regeneration (Gulen et al. 2023; Kushioka et al. 2023). However, the mechanisms governing BMM inflammaging and its deleterious impact on bone repair remain poorly understood, and effective preventive or therapeutic strategies are still lacking.

The immune system is integral to bone homeostasis and repair, with macrophages playing a central role in osteoimmunology (Schlundt et al. 2021). Following injury, macrophages undergo a highly coordinated temporal transition: classically activated M1 macrophages initiate inflammation to clear damaged tissue and pathogens, whereas alternatively activated M2 macrophages resolve inflammation and facilitate tissue regeneration (Denans et al. 2022; Zheng, Tao, et al. 2025). This phenotypic plasticity is tightly immunoregulated by local stromal cells, particularly BMSCs. However, in aging‐associated inflammatory BMM, this regulatory network is disrupted. Macrophage polarization is skewed toward a persistent pro‐inflammatory M1‐like phenotype (Clark et al. 2020; Pajarinen et al. 2019). Notably, aged BMSCs actively aggravate this imbalance through the secretion of SASP factors, which amplify M1 polarization (Yin et al. 2017; Massaro et al. 2023), thereby creating a self‐perpetuating loop that impairs bone regeneration. Consequently, elucidating how specific components of the aged BMSC secretome drive this maladaptive M1 polarization is crucial for interrupting the cycle of regenerative failure during aging.

Macrophage polarization is closely linked to cellular metabolic reprogramming, with mitochondrial integrity playing an important role (Zhao et al. 2020). Mitochondrial dysfunction, characterized by impaired oxidative phosphorylation and excessive generation of reactive oxygen species (ROS), disrupts redox homeostasis. This redox imbalance acts as a key driver of pro‐inflammatory M1‐like polarization (Lira Chavez et al. 2023; Chen et al. 2025). To maintain redox homeostasis, macrophages rely on mitophagy to selectively degrade damaged mitochondria. Efficient mitophagy alleviates oxidative stress and supports the transition toward the reparative M2 phenotype (Chen et al. 2025; Wang et al. 2023). Thus, functional mitophagy is essential for maintaining polarization balance. Conversely, impaired mitophagy disrupts these dynamics, compromising inflammation resolution and tissue repair (Tian et al. 2025; Patoli et al. 2020). Therefore, in the context of age‐related skeletal inflammation, identifying SASP factors that dysregulate macrophage mitophagy is crucial for elucidating the mechanisms underlying BMM homeostasis disruption.

Thrombospondin‐1 (Thbs1), a canonical matricellular protein and core component of the SASP, has been implicated in multiple age‐related pathologies (Murphy‐Ullrich and Suto 2018; Ramalingam et al. 2025; Porpiglia et al. 2022). Recent studies indicate that Thbs1 negatively regulates autophagy. Specifically, Thbs1 inhibition enhances protective autophagic flux across diverse cell types, suggesting that Thbs1‐mediated suppression of autophagy contributes to cellular dysfunction and disease progression (Gan et al. 2025; Catral et al. 2024; Ling et al. 2025). Mitophagy, a specialized form of autophagy critical for mitochondrial quality control (MQC), is a central regulator of macrophage polarization (Onishi et al. 2021; Van den Bossche et al. 2016). Thus, Thbs1 may impair macrophage polarization by specifically suppressing mitophagy. However, whether Thbs1 directly disrupts macrophage mitophagy in the aged BMM remains unknown. Elucidating this mechanism is essential for understanding how Thbs1 mediates aberrant crosstalk between senescent BMSCs and macrophages to disrupt bone immune homeostasis.

In this study, we screened the gene expression profiles of BMSCs derived from young and aged rats and identified Thbs1 as a key SASP factor linking BMSC senescence to macrophage dysfunction. We aim to elucidate the role and mechanism of Thbs1 in regulating macrophage mitochondrial homeostasis and function and its impact on bone regeneration in vitro and in vivo.

## 2 Methods

### 2.1 Animals

All experimental procedures were approved by the Ethics Committee of Fujian Medical University (Approval No. IACUC‐FJMU‐2025‐0076). Male Sprague–Dawley (SD) rats aged 3 months (young) and 18 months were purchased from Beijing HFK Bioscience Co. Ltd. (China). The 18‐month‐old rats were housed under specific pathogen‐free conditions with a 12‐h light/dark cycle for an additional 6 months to establish the aged model (24 months old). Rats aged 3 months (young group) and 24 months (old group) were used for subsequent experiments.

### 2.2 Isolation and Identification of BMSCs and Bone Marrow‐Derived Macrophages (BMDMs)

BMSCs were harvested from the femurs and tibias of 3‐ and 24‐month‐old SD rats following euthanasia via cervical dislocation, as previously described (Xing et al. 2023). Bone marrow cells were flushed, suspended in α‐MEM (Gibco, USA) supplemented with 10% FBS (Gibco) and 1% penicillin/streptomycin (Beyotime, China), and cultured in a humidified incubator at 37°C with 5% CO_2_. The medium was refreshed every 2 days. Adherent cells at passage 3 (P3) were used for all experiments.

BMDMs were isolated from the tibias and femurs of 3‐ and 24‐month‐old SD rats as previously described (Li et al. 2024). Briefly, bone marrow cells were cultured overnight in α‐MEM containing 10% FBS and 50 ng/mL Macrophage Colony‐Stimulating Factor (M‐CSF; PeproTech, USA). Non‐adherent cells were collected and differentiated in the presence of 50 ng/mL M‐CSF for 7 days to obtain mature BMDMs.

The immunophenotypes of BMSCs and BMDMs were verified by flow cytometry. BMSCs were incubated with antibodies against CD34, CD45, CD90, and CD105 (Abcam, USA), while BMDMs were stained with anti‐F4/80 (BioLegend, USA) at 4°C for 40 min. Analysis was performed using a BD Accuri C6 flow cytometer (BD Biosciences, USA).

### 2.3 Senescence‐Associated β‐Galactosidase (SA‐β‐Gal) Staining

SA‐β‐gal staining was performed using a staining kit (Beyotime, China) according to the manufacturer's protocol. SA‐β‐gal‐positive cells were quantified as described previously (Liu et al. 2021).

### 2.4 RT‐qPCR Analysis

Total RNA was extracted using TRIzol reagent (TaKaRa, Japan) and reverse‐transcribed into cDNA using the PrimeScript RT kit (TaKaRa, Japan). qPCR was performed using SYBR Green Mix (TaKaRa, Japan) on a Roche LightCycler 480 system (Roche, Germany). Gene expression levels were normalized to *Gapdh* using the 2^−ΔΔCt^ method. Primer sequences are listed in Table 1.

**TABLE 1:** qRT‐PCR primer sequences used in this study.

Gene name	Primer sequence (5′–3′)	
*Cdkn2a*	Forward	AGATAGACTAGCCAGGGCAGC
	Reverse	CCACTTTGACGTTGCCCATC
*Cdkn1a*	Forward	TTGTCGCTGTCTTGCACTCT
	Reverse	CTTGCAGAAGACCAATCGGC
*Trp53*	Forward	TTCGAGATGTTCCGAGAGCTG
	Reverse	GTAGACTGGCCCTTCTTGGTC
*Il1b*	Forward	GCTACCTATGTCTTGCCCGT
	Reverse	TCACACACTAGCAGGTCGTC
*Il6*	Forward	GACTTCCAGCCAGTTGCCTT
	Reverse	CTGGTCTGTTGTGGGTGGTAT
*Il10*	Forward	GTGGAGCAGGTGAAGAATGATT
	Reverse	CACGTAGGCTTCTATGCAGTTG
*Arg1*	Forward	GGACATCGTGTACATCGGCT
	Reverse	CTTCCTTCCCAGCAGGTAGC
*Tnf*	Forward	CTCAAGCCCTGGTATGAGCC
	Reverse	CTCCAAAGTAGACCTGCCCG
*Thbs1*	Forward	GAACGCCAAGTGCAACTACC
	Reverse	TCATTAGGCCAGCCGTCAAG
*Tgfbr2*	Forward	AGTGAAGAACGATTTGACCTGTT
	Reverse	GACATCCGTCTGCTTGAAGG
*Alpl*	Forward	CGTTTTCACGTTTGGTGGCT
	Reverse	ACCGTCCACCACCTTGTAAC
*Runx2*	Forward	CAGATTACAGATCCCAGGCAGAC
	Reverse	AGGTGGCAGTGTCATCATCTGAA
*Spp1*	Forward	GAGCAGTCCAAGGAGTATAAGC
	Reverse	AACTCGTGGCTCTGATGTTC
*Pink1*	Forward	GATGTGGAATATCTCGGCAGG
	Reverse	GCACAGATGAAGTGAAGGCG
*Smad3*	Forward	ACTGATCCCTCCAATTCAGAGC
	Reverse	CCAATGTAGTAGAGCCGCACA
*Stat3*	Forward	TCGACCTAGAGACCCACTCC
	Reverse	TTGGTGGTGGACGAGAACTG
*Gapdh*	Forward	ACGGCAAGTTCAACGGCACAG
	Reverse	GAAGACGCCAGTAGACTCCACGAC

### 2.5 Immunoblotting and Co‐Immunoprecipitation (Co‐IP)

Protein expression in BMSCs and BMDMs was analyzed by immunoblotting as described previously (Kim et al. 2025). Briefly, cells were lysed on ice using RIPA lysis buffer (Beyotime, China) supplemented with protease and phosphatase inhibitors. Protein concentrations were determined using a bicinchoninic acid (BCA) assay (Beyotime, China). Equal amounts of protein were separated by SDS‐PAGE, transferred to polyvinylidene difluoride membranes, and blocked with 5% non‐fat milk. The membranes were incubated overnight at 4°C with primary antibodies, followed by incubation with appropriate horseradish peroxidase (HRP)‐conjugated secondary antibodies for 1.5 h at room temperature. Protein bands were visualized using a chemiluminescence detection system. The primary antibodies used included anti‐p16 (Proteintech, China), anti‐p21 (Proteintech, China), anti‐p53 (Abclonal, China), anti‐Arg1 (HUABIO, China), anti‐TNF‐α (HUABIO, China), anti‐iNOS (Abclonal, China), anti‐IL‐6 (HUABIO, China), anti‐IL‐10 (HUABIO, China), anti‐PINK1 (HUABIO, China), anti‐Parkin (HUABIO, China), anti‐SQSTM1/p62 (Proteintech, China), anti‐microtubule‐associated protein 1 light chain 3 (LC3; Abclonal, China), anti‐TOMM20 (HUABIO, China), anti‐COX IV (HUABIO, China), anti‐TGF‐βRII (Abclonal, China), anti‐THBS1 (Proteintech, China), anti‐Smad3 (HUABIO, China), anti‐phospho‐Smad3 (CST, USA), anti‐Stat3 (HUABIO, China), anti‐phospho‐Stat3 (CST, USA), anti‐ALP (HUABIO, China), anti‐RUNX2 (Abclonal, China), anti‐OPN (HUABIO, China), and anti‐GAPDH (Proteintech, China).

For Co‐IP, cell lysates were incubated with primary antibodies against Tgfbr2 or His‐tag (for His‐Thbs1; Proteintech, China) overnight at 4°C, followed by capture with Protein A/G magnetic beads (MCE, USA). Immunoprecipitates were washed, eluted, and analyzed by immunoblotting.

### 2.6 Preparation of Conditioned Medium (CM)

CM was collected from BMSCs or BMDMs after the indicated treatments. Cells were washed and cultured in serum‐free α‐MEM for 48 h. Supernatants were collected, centrifuged at 2500 × *g* for 15 min at 4°C, and filtered through a 0.22‐μm membrane. The CM was concentrated via lyophilization, and the protein concentration was determined by BCA assay. All CM samples were normalized to a total protein concentration of 1 mg/mL prior to use in functional assays.

### 2.7 RNA‐Sequencing (RNA‐Seq)

Total RNA was extracted from young and aged BMSCs (P3). Library construction and sequencing were performed on an Illumina NovaSeq 6000 platform by Biotree Biotech Co. Ltd. (Shanghai, China). Differentially expressed genes (DEGs) were identified, and Volcano plots, heatmaps, GO, and KEGG pathway analyses were performed as previously described (Trapnell et al. 2012).

### 2.8 Small Interfering RNA (siRNA) Transfection

siRNAs targeting specific genes were obtained as follows: for BMSCs, Thbs1 and Stat3 siRNAs were from GenePharma (China) and Sangon Biotech (China), respectively; for BMDMs, Tgfbr2 and Smad3 siRNAs were from Sangon Biotech (China). The corresponding sense and antisense sequences are listed in Table 2. BMSCs were seeded and transfected with 25 nM siRNA or si‐NC using Lipofectamine RNAiMAX reagent (Invitrogen, USA) for 48 h. BMDMs were seeded and transfected under the same conditions (25 nM siRNA/si‐NC, 48 h) before further experiments.

**TABLE 2:** siRNA sequences used in this study.

si‐RNA	Primer sequence (5′–3′)	
si‐Thbs1‐1	Forward	CCACGAUAAAGAUGGUAAATT
	Reverse	UUUACCAUCUUUAUCGUGGTT
si‐Thbs1‐2	Forward	GGAGUGGACUGUAGAUAGUTT
	Reverse	ACUAUCUACAGUCCACUCCTT
si‐Tgfbr2‐1	Forward	AGAAGUCUUGCAUGAGCAA
	Reverse	UUGCUCAUGCAAGACUUCU
si‐Tgfbr2‐2	Forward	CAGAGGAGUGUAACGAUUA
	Reverse	UAAUCGUUACACUCCUCUG
si‐Smad3‐1	Forward	CGCAGAACGUGAACACCAA
	Reverse	UUGGUGUUCACGUUCUGCG
si‐Smad3‐2	Forward	GGUGCGAGAAGGCGGUCAA
	Reverse	UUGACCGCCUUCUCGCACC
si‐Stat3‐1	Forward	CGACCAGCAGUAUAGCCGA
	Reverse	UCGGCUAUACUGCUGGUCG
si‐Stat3‐2	Forward	GGAGUCCAAUGUCCUCUAU
	Reverse	AUAGAGGACAUUGGACUCC

### 2.9 Immunofluorescence (IF) Staining

Cells were fixed with 4% paraformaldehyde (PFA), permeabilized with 0.1% Triton X‐100, and blocked with 5% BSA. Samples were incubated overnight at 4°C with primary antibodies against iNOS (Abclonal, China), CD206 (HUABIO, China), TOMM20 (HUABIO, China), and LC3B (Abclonal, China), followed by fluorophore‐conjugated secondary antibodies. Nuclei were counterstained with DAPI. Images were acquired using a laser scanning confocal microscope (LSCM; ZEISS, Germany).

### 2.10 Detection of Oxidative Stress (ROS and Mitochondrial Superoxide)

Intracellular reactive oxygen species (ROS) levels were assessed using the fluorescent probe 2′,7′‐dichlorodihydrofluorescein diacetate (DCFH‐DA; Beyotime, China). For confocal imaging, adherent BMDMs grown on confocal dishes were treated under experimental conditions and incubated with 10 μM DCFH‐DA (diluted in serum‐free medium) at 37°C for 30 min in the dark. After incubation, the probe was removed by gentle PBS washing, and fluorescence images were acquired immediately using LSCM (ZEISS, Germany). For flow‐cytometric quantification, a separate batch of treated cells was harvested by gentle trypsinization, incubated with 10 μM DCFH‐DA under identical conditions, washed, resuspended in PBS, and analyzed using a flow cytometer (BD Biosciences, USA).

Mitochondrial superoxide levels were measured using MitoSOX Red (MCE, USA). For imaging, cells were co‐incubated with 100 nM MitoTracker Green (Beyotime, China) and 5 μM MitoSOX Red (MCE, USA) at 37°C for 30 min in the dark. After washing, fluorescence images were acquired using LSCM. For flow cytometry, harvested cells were stained with 5 μM MitoSOX Red under identical conditions, and red fluorescence intensity was quantified.

### 2.11 Detection of Mitochondrial Membrane Potential (MMP)

MMP was assessed using the JC‐1 fluorescent probe (MCE, USA). BMDMs were incubated with 5 μg/mL JC‐1 at 37°C for 30 min in the dark and washed. For imaging, stained cells were analyzed using LSCM to detect both red and green fluorescence. For flow cytometry, identically treated cells were harvested, stained, and analyzed. The fluorescence intensities of JC‐1 aggregates (red) and monomers (green) were measured, and the red‐to‐green ratio was calculated.

### 2.12 Flow Cytometric Analysis of Macrophage Polarization

#### 2.12.1 In Vitro Polarization Analysis

BMDMs were seeded, treated as specified, and harvested. After fixation and permeabilization, nonspecific Fc receptor binding was blocked using 2% normal rat serum. Cells were then stained with fluorescently conjugated antibodies against the M1 marker CD86 and the M2 marker CD163. Samples were assessed using a flow cytometer, and data were analyzed with FlowJo software (version 11).

#### 2.12.2 In Vivo Polarization Analysis

Single‐cell suspensions were prepared from the calvarial defect site of young, aged, and AAV9‐transduced aged rats via enzymatic digestion. After blocking with normal rat serum, cells were co‐stained with the pan‐macrophage marker F4/80 and either CD86 or CD163 to identify M1 and M2 subsets, respectively. Data acquisition and analysis followed the same procedures as for the in vitro samples.

### 2.13 Enzyme‐Linked Immunosorbent Assays (ELISA)

Thbs1 secretion from BMSCs and IL‐6 secretion from BMDMs under various experimental conditions was quantified in corresponding culture supernatants using ELISA kits (Elabscience, China) according to each manufacturer's protocols.

### 2.14 Osteogenic Differentiation Assay

For osteogenic differentiation, BMSCs subjected to different experimental treatments were cultured in osteogenic induction medium containing α‐MEM, 10% FBS, 0.1 μM dexamethasone, 10 mM β‐glycerophosphate, and 50 μg/mL ascorbic acid for up to 21 days. The medium was refreshed every 2–3 days. To assess differentiation, alkaline phosphatase (ALP) staining was performed after 7–14 days using a commercial staining kit (Beyotime, China). For matrix mineralization analysis, cells were fixed on Day 21 with 4% PFA and stained with 2% Alizarin Red S (pH 4.2; Cyagen Biosciences, USA) for 30 min at room temperature.

### 2.15 Neutralizing Antibody Assay

To neutralize specific secreted factors, neutralizing antibodies (NAbs) targeting Thbs1 or IL‐6 were added. For Thbs1 neutralization, BMDMs were treated with rThbs1 in the presence or absence of a Thbs1 NAb. For IL‐6 neutralization, an IL‐6 NAb was added to the conditioned medium (CM) from stimulated BMDMs before treating BMSCs. BMSCs were treated with CM derived from unstimulated (M0), stimulated (M1‐like STIMs), or IL‐6‐neutralized stimulated BMDMs and then harvested for signaling analysis and osteogenic evaluation.

### 2.16 Chromatin Immunoprecipitation (ChIP) Assay

ChIP was performed using a ChIP assay kit (Beyotime, China) according to the manufacturer's instructions. Chromatin from cross‐linked BMSCs and BMDMs was sonicated to 200–1000 bp fragments and incubated overnight at 4°C with antibodies against Smad3, Stat3, normal IgG (negative control), or histone H3 (positive control). Antibody–chromatin complexes were isolated using protein A/G beads. After elution and reverse cross‐linking, purified DNA was analyzed by qPCR. Primer sequences for ChIP‐qPCR are listed in Table S1, including those designed to amplify the regions containing the predicted Smad3‐binding sites in the *Pink1* promoter and the Stat3‐binding sites in the *Thbs1* promoter.

### 2.17 Subcellular Fractionation

Nuclear and cytoplasmic protein fractions were isolated using a Nuclear and Cytoplasmic Protein Extraction Kit (Beyotime, China). Cells were harvested, pelleted at 1000 × *g* for 5 min at 4°C, and processed according to the manufacturer's instructions. Fractions were stored at −80°C until analysis.

### 2.18 Rat Calvarial Defect Model and Adenovirus‐Associated Virus 9 (AAV9) Transduction

All animal procedures were approved by the institutional animal care and use committee and conducted in accordance with established guidelines (McGrath et al. 2010). Thirty‐six aged male rats (24 months old) were randomly assigned to three groups (*n* = 12 per group): the AAV9‐sh‐NC‐mScarlet control group, the AAV9‐sh‐Thbs1‐mScarlet group, and the AAV9‐sh‐Thbs1‐mScarlet + rThbs1 group. All AAV9 vectors were designed and constructed by OBiO (China). Rats received a subperiosteal injection (10^12^ vg/rat) of either AAV9‐sh‐Thbs1‐mScarlet or AAV9‐sh‐NC‐mScarlet targeted to the calvarium (sequences provided in Table S2). Four weeks after transduction, a critical‐sized calvarial defect was surgically created. Under anesthesia, a midline scalp incision was made, the periosteum was carefully elevated, and two full‐thickness 5‐mm defects were generated on either side of the sagittal suture using a trephine drill under continuous saline irrigation. A collagen membrane was placed within each defect, followed by a supplementary injection of the same AAV9 vector (10^12^ vg/rat) onto the membrane surface. In the AAV9‐sh‐Thbs1‐mScarlet + rThbs1 group, recombinant Thbs1 (rThbs1) was diluted in PBS and adsorbed onto the collagen membrane at 100 ng per defect in 50 μL during surgery. After completion of all local treatments, the skin and subcutaneous fascia were sutured. To maintain local rThbs1 exposure during healing, rThbs1 was further administered at the same dose (100 ng/defect in 50 μL) by local injection into the collagen membrane/defect region on postoperative Days 7, 14, and 21, resulting in four local administrations in total. Calcein (40 mg/kg) was administered intraperitoneally at 10 and 3 days before euthanasia. After a 1‐month healing period, rats were euthanized, and calvarial tissues were collected for analysis.

### 2.19 Isolation and Immunophenotypic Characterization of Defect‐Region Calvarial Bone‐Derived BMSCs

Defect‐region calvarial bone‐derived BMSCs were isolated 4 weeks after local AAV9 injection using a modified protocol based on previously described methods for compact bone‐ and cranial bone‐derived MSC isolation (Maeda et al. 2021; Zhu et al. 2010). Briefly, after euthanasia, the calvarial bone surrounding the defect was aseptically excised, and the periosteum, fibrous tissue, and attached soft tissues were carefully removed. The harvested bone was rinsed with sterile PBS, minced into small fragments, and digested with 1 mg/mL collagenase type II at 37°C for 40 min. After digestion, the bone fragments were washed, placed in α‐MEM supplemented with 10% FBS and 1% penicillin/streptomycin, and cultured in a humidified incubator at 37°C with 5% CO₂. Fibroblast‐like adherent cells migrating from the bone fragments were subsequently expanded and used for assessment of AAV9‐mediated mScarlet expression, verification of Thbs1 knockdown, and immunophenotypic characterization.

The immunophenotype of the isolated cells was assessed by flow cytometry. Briefly, cells were incubated with antibodies against CD34, CD45, CD90, and CD105 at 4°C for 40 min, followed by analysis using a BD Accuri C6 flow cytometer (BD Biosciences, USA).

### 2.20 Micro‐Computed Tomography (Micro‐CT) and Analysis

Rat calvariae were collected after euthanasia and fixed in 4% PFA for 24 h and scanned using a high‐resolution NEMO Micro CT system (NMC‐200, PINGSENG Healthcare, China) at 80 kV and 0.05 mA. Image reconstruction of the defect region was performed using Cruiser image acquisition software. A volume of interest corresponding to the original defect was defined for quantitative morphometric analysis. Bone volume/tissue volume (BV/TV), trabecular number (Tb.N), trabecular separation (Tb.Sp), and trabecular thickness (Tb.Th) were quantified using Avatar analysis software.

### 2.21 Histological Analysis

Calvariae were fixed in 4% PFA for 24 h and decalcified in 10% EDTA (pH 7.4) for approximately 8 weeks. Decalcified tissues were embedded in paraffin and sectioned sagittally at a thickness of 5 μm. Sections were stained with hematoxylin and eosin (H&E) for general morphology and with Masson's trichrome for collagen deposition, following standard protocols. Images were acquired using a light microscope (Nikon, Japan).

### 2.22 Immunohistochemistry (IHC) and IF

*IHC* was performed on decalcified, paraffin‐embedded femoral sections. After deparaffinization, rehydration, and antigen retrieval, endogenous peroxidase activity was quenched. Sections were blocked and incubated overnight at 4°C with primary antibodies against p16 and Thbs1. Detection was performed using an HRP‐conjugated secondary antibody and DAB chromogen, followed by hematoxylin counterstaining.

*For IF co‐staining of calvarial sections*, antigen retrieval and blocking were performed as described above. Sections were incubated overnight at 4°C with primary antibodies against OPN, F4/80, and either CD86 or CD206, or TOMM20 and LC3B. After washing, sections were incubated with fluorophore‐conjugated secondary antibodies, and nuclei were counterstained with DAPI. Fluorescent images were acquired using LSCM.

### 2.23 Statistical Analysis

Data were obtained from independent experiments or repeated measurements (*n* ≥ 3) and are presented as mean ± standard deviation (SD). Statistical significance was defined as *p* < 0.05 and denoted as follows: ns (not significant, *p* > 0.05), *p < 0.05, **p < 0.01, ***p < 0.001, and ****p < 0.0001. Comparisons between two groups were performed using unpaired, two‐tailed Student's *t*‐tests. For comparisons among multiple groups, one‐way ANOVA was performed, followed by Šídák's multiple comparisons test for preplanned pairwise comparisons when appropriate. All analyses were performed using GraphPad Prism software (version 9.0, USA).

## 3 Results

### 3.1 The Senescent BMSC Secretome Drives Macrophage Polarization Toward an M1‐Like Phenotype

**FIGURE 1:**
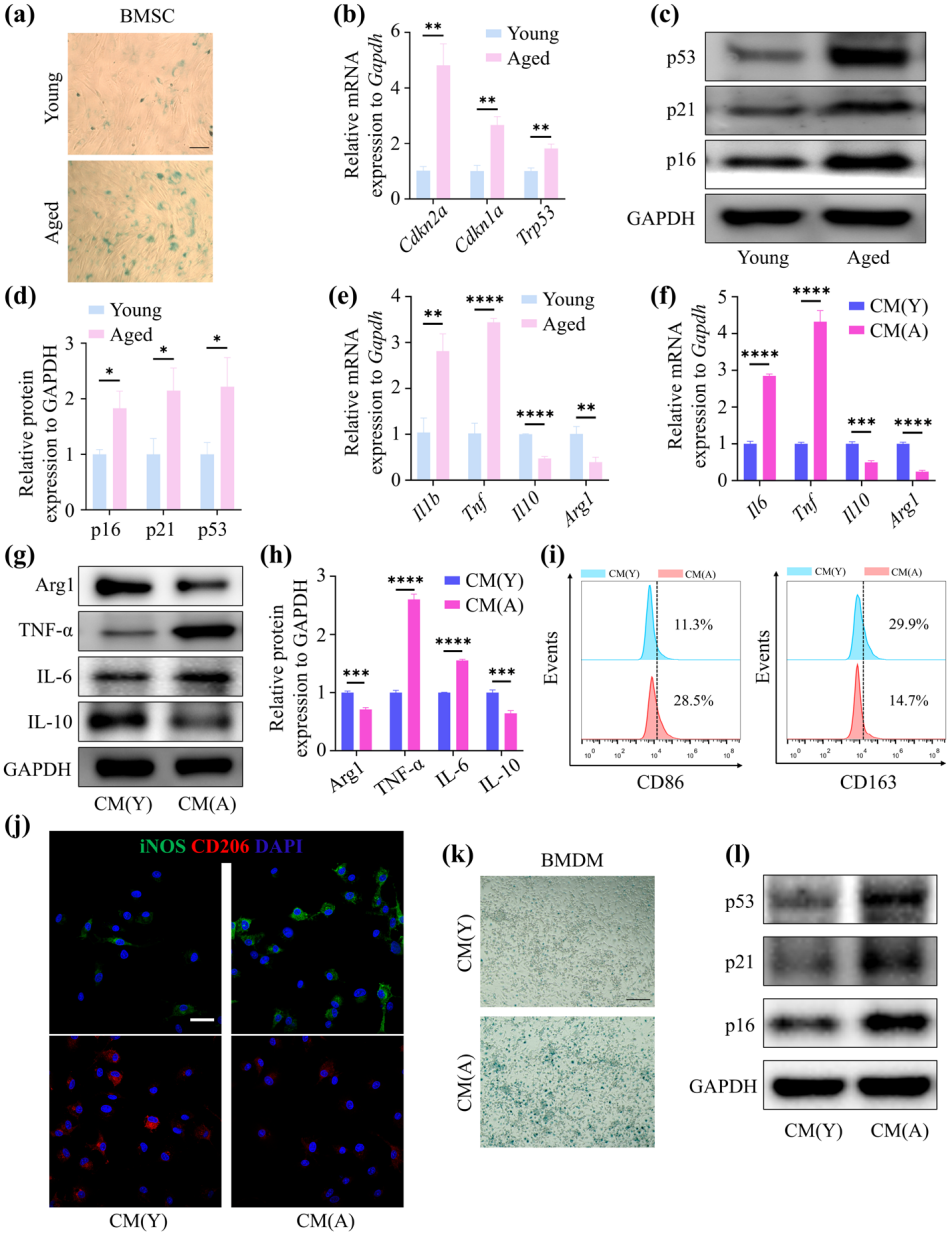
The secretome of aged BMSCs polarizes macrophages toward an M1‐like phenotype. Bone marrow‐derived macrophages (BMDMs) were treated for 48 h with conditioned medium (CM) from young (CM(Y)) or aged (CM(A)) BMSCs. (a) SA‐β‐gal staining of young and aged BMSCs (*n* = 3). Scale bar: 50 μm. (b) mRNA expression levels of senescence‐related genes (*Cdkn2a*, *Cdkn1a*, and *Trp53*) in BMSCs (*n* = 3). (c, d) Western blot analysis (c) and quantification (d) of p53, p21, and p16 protein levels in BMSCs (*n* = 3). (e) mRNA expression levels of SASP factors (*Il1b*, *Tnf*, *Il10*, and *Arg1*) in BMSCs (*n* = 3). (f) mRNA expression of M1‐associated (*Il6* and *Tnf*) and M2‐associated (*Arg1* and *Il10*) genes in BMDMs treated with CM(Y) or CM(A) (*n* = 3). (g, h) Western blot analysis (g) and quantification (h) of Arg1, TNF‐α, IL‐6, and IL‐10 protein levels in BMDMs (*n* = 3). (i) Flow cytometric analysis of M1 (CD86) and M2 (CD163) surface markers in BMDMs (*n* = 3). (j) Immunofluorescence (IF) staining of M1 (iNOS) and M2 (CD206) markers in BMDMs (*n* = 4). Scale bar: 50 μm. (k) SA‐β‐gal staining of BMDMs after treatment with CM(Y) or CM(A) (*n* = 3). Scale bar: 100 μm. (l) Western blot analysis of p53, p21, and p16 protein levels in BMDMs treated with CM(Y) or CM(A) (*n* = 3). Data are presented as mean ±  SD. Statistical significance (*p < 0.05; **p < 0.01; ***p < 0.001; ****p < 0.0001) was assessed using unpaired two‐tailed Student's *t*‐test (b, d, e, f, and h).

Inflammaging is a defining feature of the aging BMM (Li et al. 2023). To delineate its cellular basis, we characterized BMSCs and BMDMs isolated from young (3‐month) and aged (24‐month) rats. Flow cytometry confirmed a CD90^+^CD105^+^CD34^−^CD45^−^ phenotype for BMSCs (Figure S1a,b) and F4/80^+^ identity for BMDMs (Figure S1c,d). Compared with the young group, aged BMSCs exhibited a pronounced senescent phenotype, reflected by increased SA‐β‐gal positivity (Figure 1a and Figure S1e), elevated expression of senescence‐associated genes and proteins (p16, p21, and p53) (Figure 1b–d), and enhanced secretion of SASP factors (Il1b, Tnf, Il10, and Arg1) (Figure 1e). Immunohistochemical analysis further demonstrated that p16 expression in the femurs of aged rats, particularly in the trabecular bone, was higher than in the young controls (Figure S1f). Aged BMDMs also displayed intrinsic features of senescence (Figure S1g–j) and a pronounced shift toward a pro‐inflammatory M1‐like phenotype, characterized by increased expression of M1‐specific genes (*Il6* and *Tnf*) and proteins (Figure S2a–c), a higher proportion of CD86^+^ cells (Figure S2d), and enhanced iNOS signal (Figure S2e). Together, these changes constitute the cellular basis for the inflammaging that characterizes the aged bone marrow niche.

Having defined the senescent and pro‐inflammatory characteristics of BMSCs and BMDMs, we evaluated the regulatory effects of the senescent BMSC secretome on macrophage polarization. To model age‐dependent paracrine signaling, BMDMs were treated with conditioned medium from young BMSCs (CM(Y)) or aged BMSCs (CM(A)). Compared with CM(Y), CM(A) markedly upregulated M1 markers (*Il6* and *Tnf*) and downregulated M2 markers (*Arg1* and *Il10*) in macrophages (Figure 1f), with a similar trend observed at the protein level (Figure 1g,h). Flow cytometry and IF analyses revealed that CM(A) increased the proportion of CD86^+^ M1 macrophages, decreased CD163^+^/CD206^+^ M2 macrophages (Figure 1i), and was associated with stronger iNOS and weaker CD206 signals (Figure 1j). We also assessed the impact of the conditioned medium on the senescent phenotype of BMDMs. We found that CM(A) treatment promoted BMDM senescence, as indicated by an increase in SA‐β‐gal staining positivity and upregulation of senescence‐associated proteins p16, p21, and p53 (Figure 1k,l). These findings demonstrate that the senescent BMSC secretome is sufficient to promote M1 polarization and cellular senescence in macrophages, thereby amplifying the inflammatory milieu characteristic of aged bone marrow.

### 3.2 The Senescent BMSC Secretome Impairs Mitochondrial Function and Mitophagy in Macrophages

Macrophage polarization is closely linked to mitochondrial remodeling, and both mitochondrial dysfunction and impaired mitophagy are recognized drivers of M1 polarization (Liu et al. 2021). Building on our finding that CM(A) promotes M1 skewing, we examined how senescent BMSC secretome affects mitochondrial function in macrophages. Total intracellular ROS were measured using the DCF‐DA probe, and mitochondrial ROS were assessed with MitoSOX. Compared with CM(Y), CM(A) significantly elevated both total and mitochondrial ROS signals (Figure S3a,b), consistent with flow cytometric quantification (Figure S3c,d). MMP was evaluated using JC‐1 staining and flow cytometry. CM(A) induced substantial membrane depolarization, indicated by a shift from red JC‐1 aggregates to green monomers (Figure S3e–g), suggesting impaired mitochondrial function. These findings suggest that senescent BMSC secretome disrupts mitochondrial redox homeostasis and membrane potential.

Mitophagy is a key mechanism for selectively degrading damaged mitochondria and maintaining MQC (Wang et al. 2023). Given this CM(A)‐induced mitochondrial dysfunction, we examined mitophagy. Dual IF staining for LC3B and the mitochondrial outer membrane protein TOMM20 revealed a significant reduction in LC3B–TOMM20 colocalization in CM(A)‐treated macrophages (Figure S3h), indicative of impaired mitophagy flux. Mitophagy is primarily mediated by the ubiquitin‐dependent PINK1/Parkin pathway, one of the most extensively characterized mechanisms of MQC (Zheng, Li, et al. 2025). Western blot analysis further demonstrated that CM(A) decreased PINK1 and Parkin protein levels, increased the accumulation of the autophagy substrate p62, reduced the LC3‐II/I ratio, and caused the accumulation of the mitochondrial proteins COXIV and TOMM20 (Figure S3i). Together, these findings indicate that the senescent BMSC secretome suppresses PINK1/Parkin‐mediated mitophagy, resulting in mitochondrial dysfunction.

### 3.3 Senescent BMSC Secretome‐Induced M1 Polarization Is Mediated by the Suppression of Mitophagy

**FIGURE 2:**
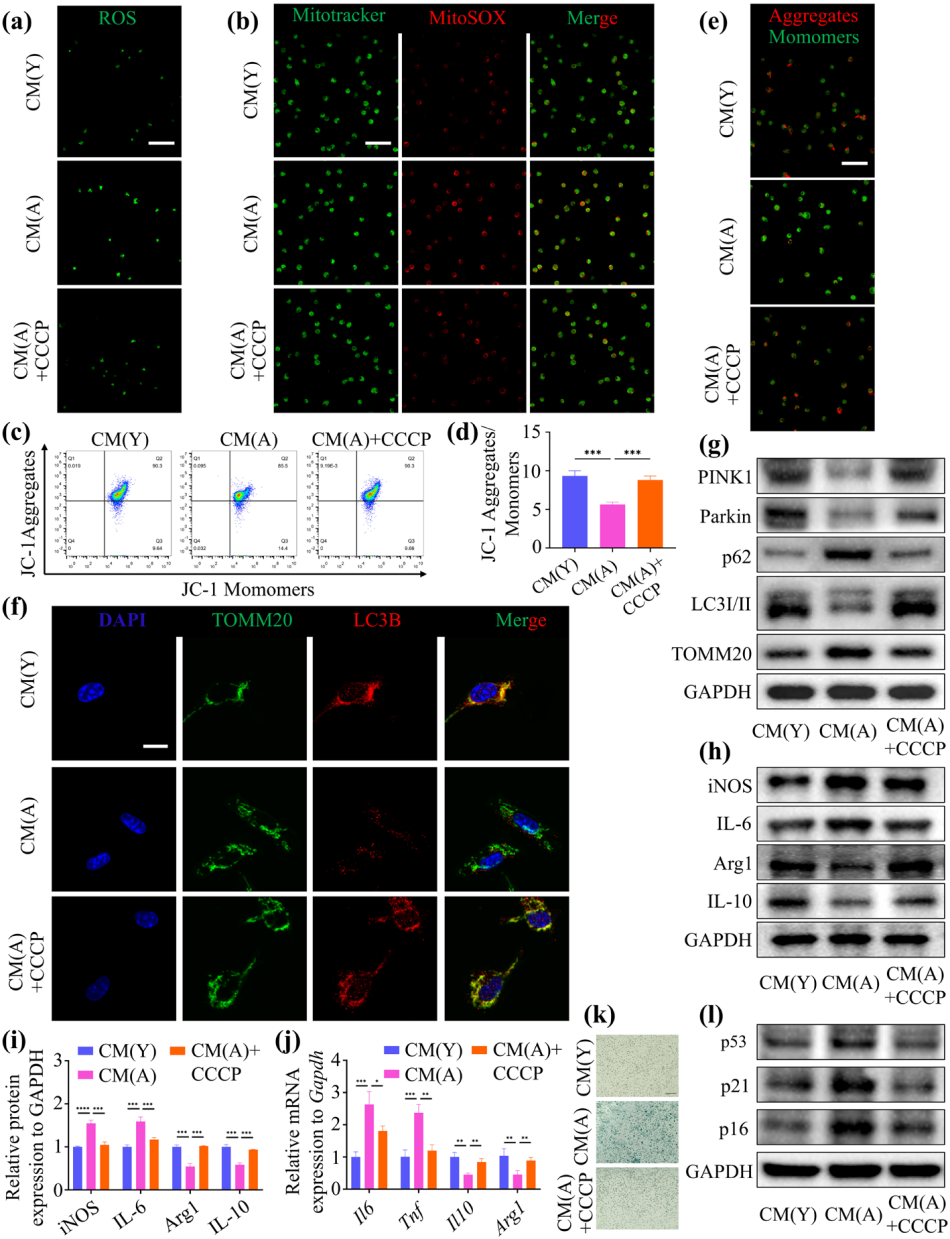
CCCP rescues mitochondrial dysfunction, restores mitophagy, and attenuates M1 polarization and senescence in macrophages. BMDMs were treated with CM(Y) or CM(A) for 48 h, with or without the mitophagy activator CCCP (10 μM). (a) Representative images of DCF‐DA staining (green) revealing total ROS in BMDMs (*n* = 3). Scale bar: 50 μm. (b) Representative images of Mitotracker (green) and MitoSOX (red) co‐staining in BMDMs (*n* = 3). Scale bar: 50 μm. (c, d) Flow cytometric analysis of mitochondrial membrane potential (MMP) (aggregate‐to‐monomer ratio) in BMDMs (*n* = 3). (e) Mitochondrial membrane potential assessed by JC‐1 staining (aggregates, red; monomers, green) in BMDMs (*n* = 3). Scale bar: 50 μm. (f) Representative fluorescence images of TOMM20 (green) and LC3B (red) double staining in BMDMs (*n* = 3). Scale bar: 10 μm. (g) Western blot analysis of PINK1, Parkin, p62, LC3I/II, and TOMM20 protein levels in BMDMs (*n* = 3). (h, i) Western blot analysis (h) and quantification (i) of iNOS, IL‐6, Arg1, and IL‐10 protein levels in BMDMs (*n* = 3). (j) mRNA expression levels of M1‐related (*Il6* and *Tnf*) and M2‐related (*Il10* and *Arg1*) genes in BMDMs (*n* = 3). (k) SA‐β‐gal staining of BMDMs (*n* = 3). Scale bar: 100 μm. (l) Western blot analysis of p53, p21, and p16 protein levels in BMDMs (*n* = 3). Data are presented as mean ± SD. Statistical significance (*p < 0.05; **p < 0.01; ***p < 0.001; ****p < 0.0001) was assessed using one‐way ANOVA with Šídák's multiple comparisons test (d, i, j).

Impaired mitophagy is a defining feature of M1 polarization (Esteban‐Martínez et al. 2017). To further assess the contribution of mitophagy to CM(A)‐induced M1 skewing, we used the mitochondrial uncoupler carbonyl cyanide m‐chlorophenyl hydrazone (CCCP) to activate the PINK1/Parkin‐mediated mitophagy (Ma et al. 2021) in the presence of CM(A) and evaluated mitochondrial function and mitophagy in macrophages. DCF‐DA and MitoSOX staining revealed that, compared with CM(A) alone, CCCP co‐treatment significantly decreased total intracellular ROS and mitochondrial ROS (Figure 2a,b). Furthermore, CCCP partially reversed CM(A)‐induced MMP loss, reflected by an increased ratio of red JC‐1 aggregates to green monomers (Figure 2c–e), indicating a partial recovery of mitochondrial function. To confirm mitophagy reactivation, dual IF staining demonstrated that CCCP co‐treatment increased LC3B–TOMM20 colocalization puncta (Figure 2f). Consistently, Western blot analysis showed that CCCP increased PINK1 and Parkin protein levels, elevated the LC3‐II/I ratio, and concomitantly reduced p62 and TOMM20 levels (Figure 2g), consistent with enhanced mitophagy‐related activity and the clearance of damaged mitochondria.

We next investigated how restoring mitophagy influences macrophage polarization. As observed previously, CM(A) promoted M1 polarization, whereas mitophagy activation by CCCP in the presence of CM(A) significantly reduced the expression of M1‐specific genes (*Il6* and *Tnf*) and proteins (iNOS and IL‐6) (Figure 2h–j). During aging, macrophages spontaneously shift toward an M1‐like phenotype, and macrophage senescence is closely linked to mitochondrial dysfunction and impaired mitophagy (Minhas et al. 2019; Zhong et al. 2022). We therefore examined the impact of CCCP‐induced mitophagy activation on macrophage senescence. SA‐β‐gal staining revealed that the senescent phenotype induced by CM(A) was partially reversed by CCCP (Figure 2k). Furthermore, the expression of senescence‐associated proteins, including p16, p21, and p53, was reduced compared with the CM(A) group (Figure 2l). Together, these findings demonstrate that the suppression of mitophagy contributes to CM(A)‐induced M1 macrophage polarization. Restoring mitophagy through CCCP treatment alleviates mitochondrial dysfunction and cellular senescence, leading to a reduction in M1 polarization.

### 3.4 BMSC‐Derived Thbs1 Suppresses Macrophage Mitophagy to Drive M1 Polarization

**FIGURE 3:**
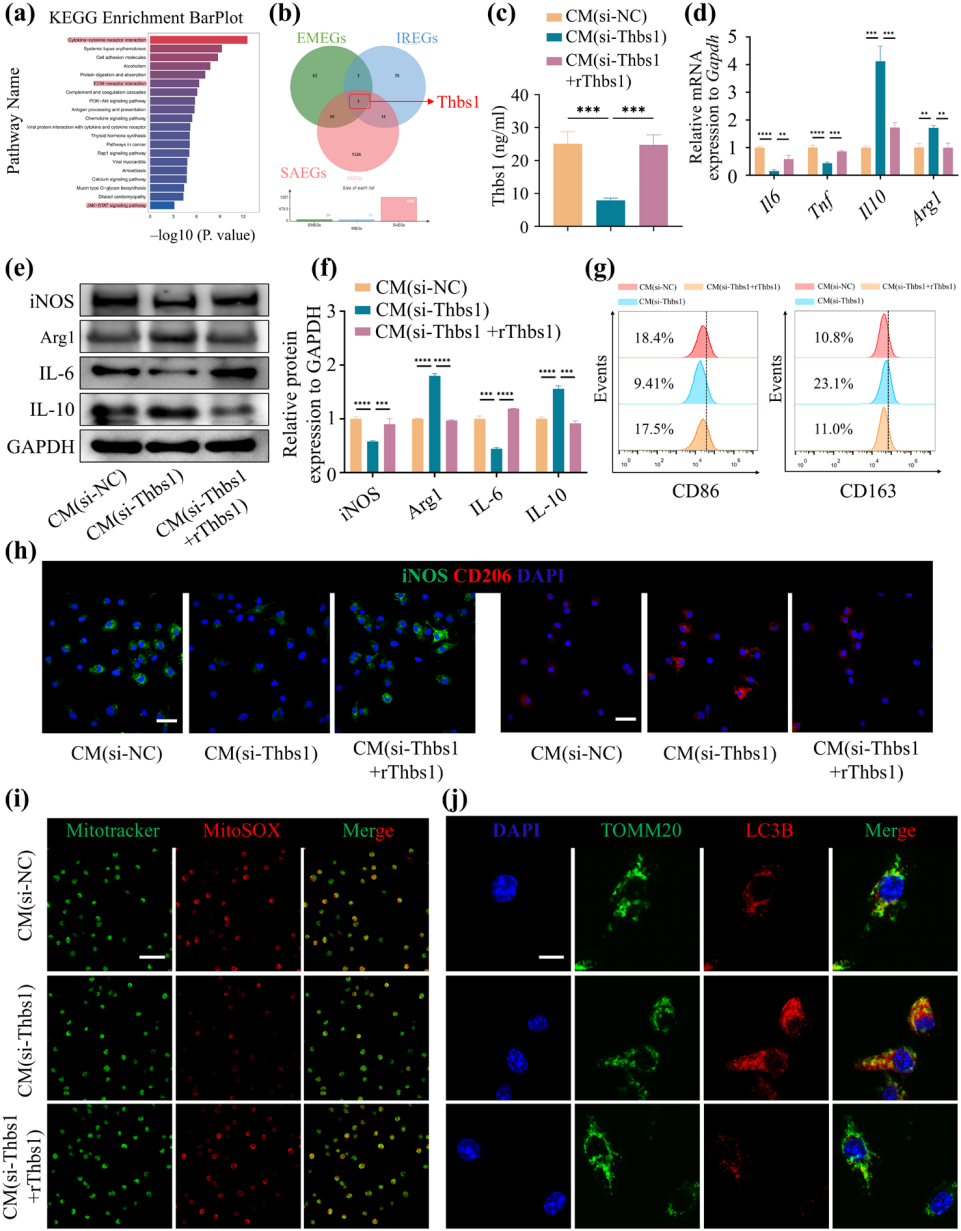
Thbs1 from senescent BMSCs impairs macrophage mitophagy to drive M1 polarization. BMDMs were treated for 48 h with CM from aged BMSCs subjected to Thbs1 knockdown (si‐Thbs1), control siRNA (si‐NC), or rescued with recombinant Thbs1 (si‐Thbs1 + rThbs1; 25 ng/mL). (a) KEGG pathway enrichment analysis of differentially expressed genes in young and aged BMSCs (*n* = 4). (b) Venn diagram illustrating the overlap of differentially expressed genes associated with extracellular matrix (EMEGs), immune response (IREGs), and senescence (SAEGs), identifying Thbs1 as a common candidate (*n* = 4). (c) Thbs1 concentrations in CM from BMSCs transfected with si‐NC or si‐Thbs1, with or without rThbs1 add‐back, were measured by ELISA (*n* = 3). (d) mRNA expression levels of M1‐ and M2‐associated genes in BMDMs under the indicated conditions (*n* = 3). (e, f) Western blot analysis (e) and quantification (f) of iNOS, Arg1, IL‐6, and IL‐10 protein expression in BMDMs (*n* = 3). (g) Flow cytometric analysis of M1 (iNOS) and M2 (CD206) markers in BMDMs (*n* = 3). (h) IF staining of iNOS (green) and CD206 (red) in BMDMs (*n* = 4). Scale bar: 25 μm. (i) Mitochondrial ROS levels assessed by MitoSOX staining in BMDMs (*n* = 3). Scale bar: 50 μm. (j) Representative IF images revealing TOMM20 (green) and LC3B (red) double staining in BMDMs (*n* = 3). Scale bar: 10 μm. Data are presented as mean ± SD. Statistical significance (**p < 0.01; ***p < 0.001; ****p < 0.0001) was assessed using one‐way ANOVA with Šídák's multiple comparisons test (c, d, f).

After establishing an negative correlation between macrophage M1 polarization and mitophagy, we sought to identify the upstream drivers within the senescent BMSC secretome. RNA sequencing of young and aged BMSCs, followed by integrated bioinformatics analysis, revealed extensive transcriptional rewiring. Volcano plots and heatmaps demonstrated significant differential gene expression (Figure S4a,b). GO/KEGG enrichment analyses highlighted pathways related to extracellular matrix (ECM), ECM−receptor interaction, cytokine–cytokine receptor interaction, immune response, and inflammatory response (Figure 3a and Figure S4c), consistent with SASP‐mediated microenvironmental remodeling. Given the known immunoregulatory crosstalk between BMSCs and macrophages, we focused on secreted proteins. To identify relevant candidates, we intersected 1357 senescence‐associated genes (SAEGs) from the GeneCards database (relevance score > 5) (Liu et al. 2023) with our ECM and immune regulation gene sets (Table S3). This analysis identified Thbs1 as a prominently upregulated candidate in aged BMSCs (Figure 3b and Figure S4d). Thbs1 induction was confirmed at both the mRNA (Figure S4e) and cellular protein (Figure S4f,g) levels in cultured BMSCs. ELISA quantification of CM further showed that aged BMSCs secreted significantly higher levels of Thbs1 than young BMSCs (22.08 ± 1.87 vs. 7.49 ± 0.39 ng/mL; Figure S4h). Additionally, IHC confirmed increased Thbs1 expression in aged bone tissue (Figure S4i).

To define the role of Thbs1, we employed loss‐ and gain‐of‐function approaches. Thbs1 knockdown in aged BMSCs using siRNA (Figure S5a–d) markedly diminished the pro‐inflammatory activity of their secretome. ELISA quantification showed that Thbs1 knockdown reduced Thbs1 concentration in CM from 25.11 ± 3.64 ng/mL in CM(si‐NC) to 7.91 ± 0.67 ng/mL in CM(si‐Thbs1), whereas rThbs1 add‐back restored Thbs1 levels to 24.76 ± 2.99 ng/mL in CM(si‐Thbs1 + rThbs1) (Figure 3c). Macrophages treated with CM from Thbs1‐knockdown BMSCs exhibited reduced expression of M1 markers (IL‐6 and TNF‐α) and increased expression of M2 markers (IL‐10 and Arg1) (Figure 3d–f). Flow cytometry and IF corroborated these findings, indicating decreased CD86^+^/iNOS^+^ M1 macrophages and increased CD163^+^/CD206^+^ M2 macrophages (Figure 3g,h), whereas the add‐back of recombinant Thbs1 (rThbs1) reversed these phenotypes (Figure 3d–h). To further validate the role of Thbs1 in macrophage polarization, we treated macrophages with rThbs1, with or without Thbs1 neutralizing antibody (NAb). Flow cytometry and IF analysis revealed that Thbs1 treatment promoted M1 polarization, which was reversed by Thbs1 NAb addition (Figure S6a,b). These results establish Thbs1 as necessary for the M1‐skewing activity of the senescent BMSC secretome.

We further investigated whether Thbs1 drives M1 polarization by disrupting MQC. Thbs1 knockdown in aged BMSCs attenuated the CM(A)‐induced increase in total and mitochondrial ROS (Figure 3i and Figure S7a–c) and partially restored MMP (Figure S7d–f) in BMDMs. Dual IF staining revealed a marked increase in LC3B–TOMM20 colocalization after Thbs1 knockdown (Figure 3j). Consistently, Western blot analysis confirmed higher PINK1 and Parkin expression, an increased LC3‐II/I ratio, and reduced levels of p62 and mitochondrial proteins (Figure S7g). Importantly, these protective effects were partially reversed by rThbs1 add‐back. Collectively, these findings indicate that BMSC‐derived Thbs1 promotes macrophage M1 polarization, at least in part, by inhibiting PINK1/Parkin‐mediated mitophagy and inducing mitochondrial dysfunction.

### 3.5 Senescent BMSC‐Derived Thbs1 Activates TGF‐β/Smad3 Signaling to Suppress Mitophagy and Drive Macrophage M1 Polarization

The canonical TGF‐β/Smad3 pathway is a key regulator of macrophage polarization, and Thbs1 has been implicated in TGF‐β activation (Vanhoutte et al. 2024). To define the molecular mechanism, we first examined the interaction between Thbs1 and the Tgfbr2. Co‐IP assays confirmed an interaction between endogenous Thbs1 and Tgfbr2 (Figure S8a,b). Functionally, CM(A) stimulation markedly increased Smad3 phosphorylation (p‐Smad3) in macrophages, whereas Thbs1 knockdown in aged BMSCs significantly prevented this response (Figure S8c). To confirm the specificity of the Tgfbr2/Smad3 signaling, we combined recombinant protein stimulation with receptor knockdown and pharmacological interventions. rThbs1 alone robustly induced Smad3 phosphorylation, and this effect was abolished (Figure S8g,h) when Tgfbr2 was successfully knocked down (Figure S8d–f). In parallel, the M1‐like phenotype triggered by rThbs1—characterized by elevated IL‐6 and TNF‐α, reduced Arg1 and IL‐10 (Figure S9a–c), expansion of CD86^+^/iNOS^+^ M1 macrophages, and a decreased proportion of CD163^+^/CD206^+^ M2 macrophages (Figure S9d,e)—was markedly attenuated in Tgfbr2‐deficient cells. Furthermore, pharmacological inhibition of Smad3 using the Smad3‐specific inhibitor SIS3 effectively suppressed rThbs1‐induced Smad3 phosphorylation (Figure S10a) and partially reversed the M1‐like phenotype while restoring M2‐associated features (Figure S10b–e). Collectively, these observations indicate that Thbs1 drives macrophage M1 polarization by activating TGF‐β/Smad3 signaling.

We next investigated whether TGF‐β/Smad3 signaling mediates Thbs1‐induced mitochondrial defects. Previous studies have identified this pathway as an essential regulator of mitochondrial function and mitophagy (Yuan et al. 2025; Yan et al. 2025). We observed that direct stimulation of macrophages with rThbs1 recapitulated the deleterious effects of the senescent secretome, inducing pronounced oxidative stress (Figure S11a–d), MMP loss (Figure S11e–g), and impaired mitophagy (Figure S12a,b). Combined genetic and pharmacologic interventions partially reversed these changes: *Tgfbr2* knockdown or Smad3 inhibition with SIS3 reduced ROS accumulation (Figures S11a–d and S13a–d) and MMP loss (Figures S11e–g and S13e), restored PINK1/Parkin levels, and improved mitophagy flux markers (Figures S12a,b and S13f). Together, these findings indicate that Thbs1 from senescent BMSCs activates macrophage TGF‐β/Smad3 signaling to selectively inhibit PINK1/Parkin‐mediated mitophagy, thereby inducing mitochondrial dysfunction and promoting M1 polarization.

### 3.6 Thbs1‐Induced Smad3 Nuclear Translocation Represses *Pink1* to Inhibit Macrophage Mitophagy

**FIGURE 4:**
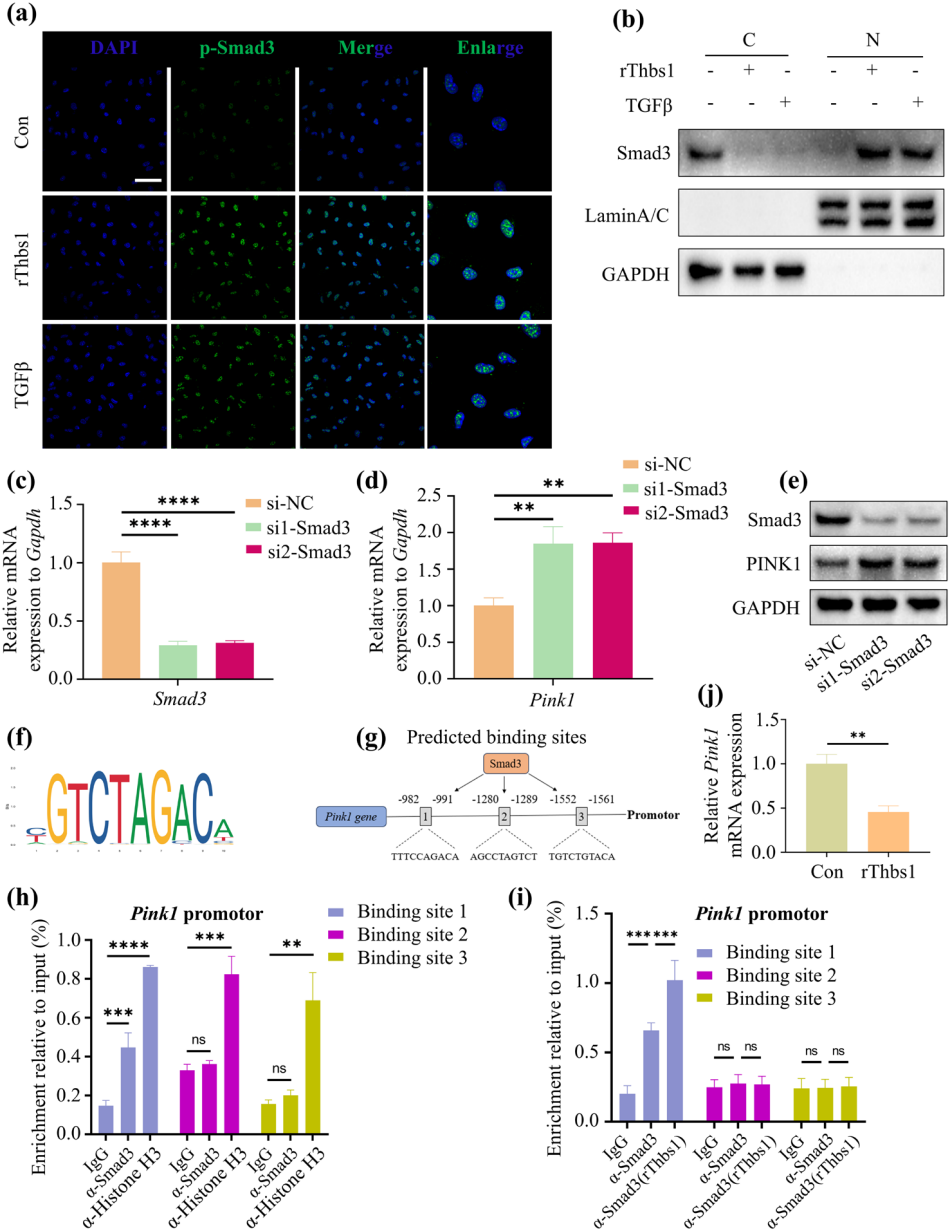
Thbs1 promotes Smad3 nuclear translocation to repress *Pink1* and inhibit macrophage mitophagy transcriptionally. (a) IF images illustrating p‐Smad3 (green) localization in BMDMs treated with rThbs1, TGF‐β, or vehicle control. Nuclei were counterstained with DAPI (blue) (*n* = 4). Scale bar: 25 μm. (b) Western blot analysis of Smad3 distribution in nuclear (N) and cytoplasmic (C) fractions of BMDMs treated with rThbs1 or TGF‐β. GAPDH and Lamin A/C served as cytoplasmic and nuclear loading controls, respectively (*n* = 3). (c, d) mRNA expression levels of *Smad3* and *Pink1* in BMDMs transfected with control siRNA (si‐NC) or Smad3‐targeting siRNA (si‐Smad3) (*n* = 3). (e) Western blot analysis of Smad3 and PINK1 protein levels in BMDMs (*n* = 3). (f) Bioinformatics prediction of the Smad3 binding motif within the *Pink1* promoter region. (g) Schematic representation of three predicted Smad3 binding sites in the *Pink1* promoter (−982 to −991, −1280 to −1289, and −1552 to −1561), identified using the JASPAR database. (h) ChIP‐PCR analysis of Smad3 occupancy at the *Pink1* promoter in BMDMs (*n* = 3). (i) ChIP‐PCR analysis revealing enhanced Smad3 binding to site 1 of the *Pink1* promoter following rThbs1 treatment (*n* = 3). (j) mRNA expression levels of Pink1 in BMDMs treated with rThbs1 (*n* = 3). Data are presented as the mean ± SD. Statistical significance (**p < 0.01; ***p < 0.001; ****p < 0.0001; ns, not significant) was assessed using unpaired two‐tailed Student's *t*‐test (j), one‐way ANOVA with Dunnett's test (c, d, and h), or one‐way ANOVA with Šídák's multiple comparisons test (i).

Having established that Thbs1 suppresses PINK1/Parkin‐mediated mitophagy via the TGF‐β/Smad3 pathway, we sought to delineate the underlying molecular mechanism. Given that Smad3 is a canonical TGF‐β‐responsive transcription factor (Miyazawa et al. 2024) and our data suggested an inverse relationship between p‐Smad3 and PINK1 levels, we predicted that Smad3 might directly repress *Pink1* transcription. To validate this hypothesis, we first examined Smad3 subcellular distribution after rThbs1 stimulation. Similar to TGF‐β, stimulation with rThbs1 enhanced Smad3 phosphorylation and nuclear accumulation, as confirmed by both IF and nuclear‐cytoplasmic fractionation analyses (Figure 4a,b). We next asked whether nuclear Smad3 could transcriptionally regulate *Pink1*. Smad3 knockdown in macrophages significantly increased *Pink1* mRNA (Figure 4c,d) and protein levels (Figure 4e), supporting a repressive role. Through bioinformatic analysis of the *Pink1* promoter using the JASPAR database, we identified three potential Smad3‐binding sites (Figure 4f,g). ChIP assays revealed specific enrichment of Smad3 at site 1 (−982 bp), whereas the other sites exhibited no enrichment (Figure 4h). Notbly, rThbs1 treatment further enhanced Smad3 occupancy at site 1 (Figure 4i), accompanied by the downregulation of *Pink1* mRNA (Figure 4j). Together, these data demonstrate that Thbs1‐activated Smad3 translocates to the nucleus and binds to a conserved region within the *Pink1* promoter to repress its transcription, providing a mechanistic explanation for how Thbs1 impairs mitophagy and exacerbates mitochondrial dysfunction.

### 3.7 Senescent BMSC‐Derived Thbs1 Suppresses Osteogenesis via an M1 Macrophage–IL‐6/JAK/STAT3 Feedback Loop

We previously showed that senescent BMSCs drive M1 polarization via Thbs1. To link this inflammatory state to aging‐associated defects in bone repair, we hypothesized that senescent Thbs1‐induced M1‐like macrophages (M1‐like STIMs) feed back on BMSCs to inhibit osteogenic differentiation. To test this hypothesis, we first investigated the relationship between the aged microenvironment, impaired bone regeneration, and M1 polarization in vivo using a rat calvarial defect model. As illustrated in Figure S14a,b, micro‐CT analysis revealed markedly diminished bone formation in aged rats, reflected by reduced BV/TV, Tb.Th, and Tb.N, and increased Tb.Sp. H&E and Masson's trichrome staining further demonstrated sparse new bone formation and collagen deposition (Figure S14c,d), while IF revealed reduced osteopontin (OPN) expression within the defect area (Figure S14e). In parallel, flow cytometry and IF analyses revealed a higher proportion of F4/80^+^CD86^+^ M1 macrophages and relatively fewer CD163^+^/CD206^+^ M2 macrophages in aged defects (Figure S14f–h), indicating that compromised regeneration is associated with a skewed M1‐polarized immune landscape.

**FIGURE 5:**
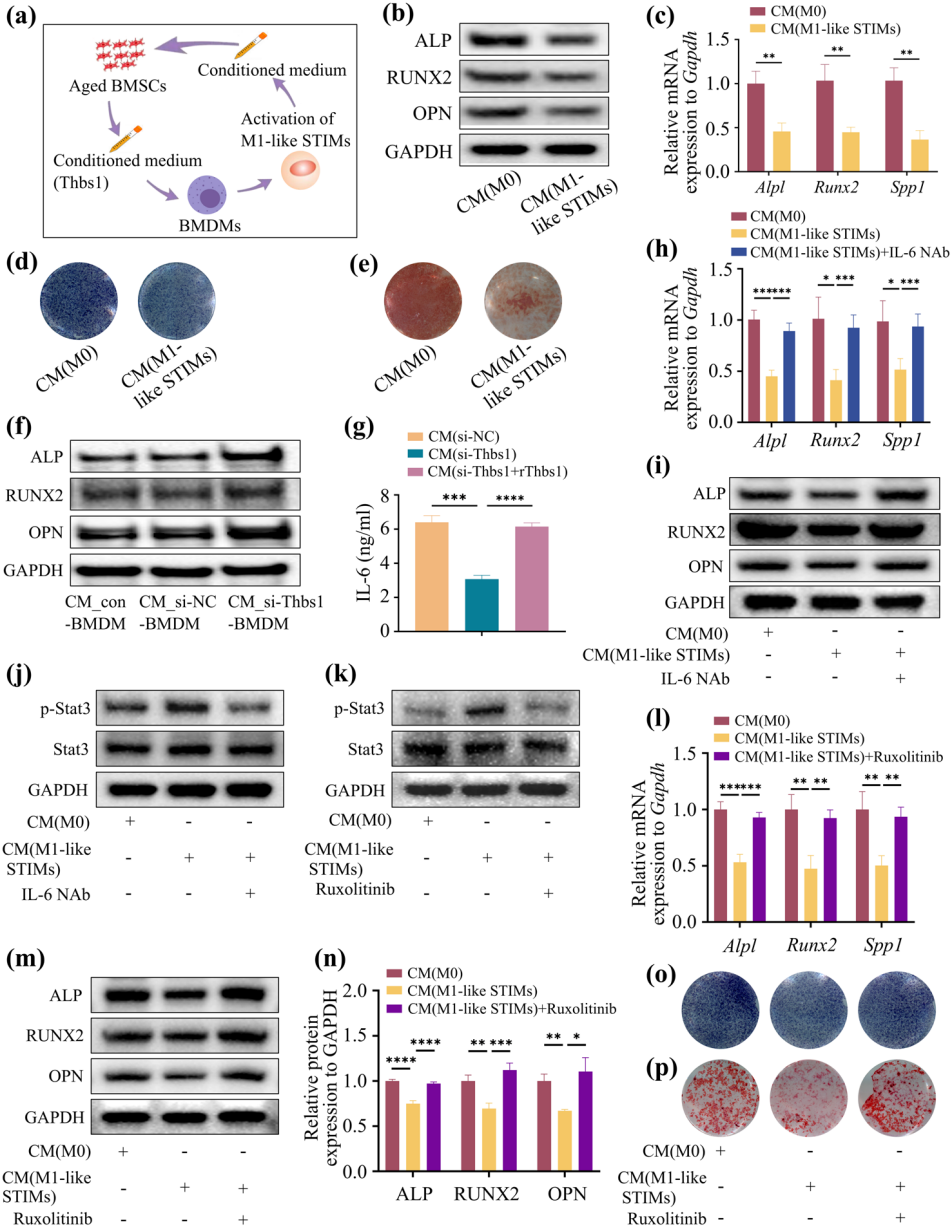
Thbs1 secreted by aged BMSCs inhibits osteogenesis through an M1 macrophage‐driven IL‐6/JAK/STAT3 feedback loop. (a) Schematic illustration of the Thbs1‐mediated bidirectional regulatory loop between aged BMSCs and BMDMs. (b) Western blot analysis of osteogenic markers (ALP, RUNX2, and OPN) in BMSCs treated with CM from unpolarized (M0) or M1‐like STIMs (*n* = 3). (c) qPCR analysis of osteogenic genes (*Alpl*, *Runx2*, and *Spp1*) in BMSCs treated with CM from unpolarized (M0) or M1‐like STIMs (*n* = 3). (d, e) Representative alkaline phosphatase (ALP) (d) and Alizarin Red S staining (ARS) (e) images of BMSCs treated with CM from unpolarized (M0) or M1‐like STIMs (*n* = 3). (f) Western blot analysis of osteogenic markers in BMSCs treated with CM from control BMDMs, si‐NC‐CM BMDMs, or si‐Thbs1‐CM BMDMs (*n* = 3). (g) ELISA analysis of IL‐6 secretion in BMDMs treated with CM(si‐Thbs1) from aged BMSCs, with or without rThbs1 add‐back (*n* = 3). (h, i) qPCR (h) and Western blot (i) analyses of osteogenic gene markers in BMSCs treated with CM(M1‐like STIMs) in the presence of IL‐6‐neutralizing antibody (IL‐6 NAb) (*n* = 3). (j) Western blot analysis of p‐Stat3 and total Stat3 in BMSCs treated with CM(M1‐like STIMs) and IL‐6 NAb (*n* = 3). (k) Western blot analysis of p‐Stat3/Stat3 signaling in BMSCs treated with CM(M1‐like STIMs) and the JAK inhibitor ruxolitinib (*n* = 3). (l) qPCR analysis of osteogenic gene expression in BMSCs treated with CM(M1‐like STIMs) and ruxolitinib (*n* = 3). (m, n) Western blot analysis (m) and quantification (n) of osteogenic markers under ruxolitinib treatment (*n* = 3). (o, p) Representative ALP (o) and ARS (p) staining images of BMSCs treated with CM(M0), CM(M1‐like STIMs), or CM(M1‐like STIMs) + ruxolitinib (*n* = 3). Data are presented as the mean ± SD. Statistical significance (*p < 0.05; **p < 0.01; ***p < 0.001; ****p < 0.0001) was assessed using unpaired two‐tailed Student's *t*‐test (c) or one‐way ANOVA with Šídák's multiple comparisons test (g, h, l, n).

To further explore this interaction, we established an in vitro feedback model by collecting CM from M1‐like STIMs and applying it to BMSCs (Figure 5a). This medium markedly inhibited BMSC osteogenic differentiation, as evidenced by reduced expression of osteogenic genes (*Alpl*, *Runx2*, and *Spp1*) and proteins (Figure 5b,c), decreased ALP activity (Figure 5d), and impaired mineralization (Figure 5e). These findings indicate that M1‐like STIMs function as osteogenesis‐inhibitory effector cells, mediating a negative feedback loop from senescent BMSCs to macrophages and back to BMSCs.

To confirm the role of Thbs1 in this feedback loop, we knocked down Thbs1 in senescent BMSCs and treated BMDMs with CM harvested from these cells. ELISA assays showed that IL‐6 levels in BMDM‐derived CM were markedly reduced in the CM_si‐Thbs1‐BMDM group (1.39 ± 0.11 ng/mL) compared with the CM_con‐BMDM group (5.99 ± 0.32 ng/mL) and CM_si‐NC‐BMDM group (5.87 ± 0.47 ng/mL) (Figure S15a), indicating that senescent BMSC‐derived Thbs1 promotes IL‐6 production by macrophages. Consistently, the CM harvested from these STIMs (CM_si‐Thbs1‐BMDM) largely reversed the osteoinhibitory phenotype when applied to BMSCs, as evidenced by increased mRNA and protein expression of osteogenic markers (Figure 5f and Figure S15b) and partially restored ALP activity and mineralization (Figure S15c,d). These results identify Thbs1 as a key mediator in the senescent BMSC secretome that confers osteoinhibitory properties on macrophages.

To identify the macrophage‐derived effectors responsible for this inhibitory effect, we reanalyzed the transcriptomes of young and aged BMSCs. This analysis revealed substantial remodeling of the JAK–STAT pathway in aged BMSCs, including the upregulation of IL‐6 receptor (*Il6r*) and downregulation of SOCS family negative regulators (Figure 3a and Table S4), suggesting that aged BMSCs are hypersensitized to IL‐6 signaling. We therefore investigated the functional role of IL‐6, a key ligand driving JAK–STAT pathway activation in aged BMSCs. ELISA assays revealed that BMDMs stimulated with CM from Thbs1‐deficient senescent BMSCs produced significantly less IL‐6 (3.07 ± 0.22 ng/mL) than controls (6.40 ± 0.39 ng/mL); this reduction was reversed by rThbs1 supplementation (6.15 ± 0.21 ng/mL) (Figure 5g), indicating that senescent BMSC‐derived Thbs1 functions upstream to regulate BMDM IL‐6 production. Neutralization of IL‐6 in M1‐like STIMs CM effectively relieved suppression of BMSC osteogenic gene (Figure 5h) and protein expression (Figure 5i and Figure S16a) and partially restored ALP activity (Figure S16b) and mineralization (Figure S16c). Consistently, ELISA assays revealed that detectable IL‐6 levels were markedly increased in M1‐like STIMs CM compared with M0 CM (5.81 ± 0.40 vs. 1.47 ± 0.08 ng/mL), whereas supplementation with IL‐6 NAb reduced detectable IL‐6 levels to 2.34 ± 0.21 ng/mL (Figure S16d). These findings identify IL‐6 as a key downstream osteogenesis‐inhibitory effector of Thbs1. We next applied the JAK/STAT3 pathway inhibitor ruxolitinib. Western blot analysis demonstrated that Stat3 phosphorylation induced by M1‐like STIMs CM was markedly reduced by either IL‐6 NAb (Figure 5j) or ruxolitinib (Figure 5k). Moreover, ruxolitinib also reversed osteogenic inhibition (Figure 5l–p). Collectively, these results demonstrate that Thbs1‐reprogrammed macrophages secrete IL‐6, which activates the JAK/STAT3 pathway in BMSCs to suppress osteogenesis, establishing a pathogenic feedback loop.

### 3.8 Stat3 Directly Drives *Thbs1* Transcription to Sustain a Self‐Amplifying Pro‐Inflammatory Loop in Senescent BMSCs

Having defined a senescent BMSC–M1‐like macrophage–IL‐6/JAK/STAT3 paracrine feedback circuit, we next examined whether this pathway sustains inflammation through transcriptional regulation. Comprehensive transcriptomic analysis of BMSCs revealed that, among genes at the intersection of aging‐ and inflammation‐related GO terms, including regulation of cell cycle, immune response, inflammatory response, and chronic inflammatory response, *Thbs1* was the only gene consistently upregulated (Figure S17a and Table S5), suggesting a nodal role in linking inflammatory signaling to BMSC functional decline. To elucidate the transcriptional basis of *Thbs1* induction, we used the JASPAR database to predict transcription factor binding sites. Among candidate transcription factors, the *Thbs1* promoter exhibited the highest predicted binding affinity for Stat3 (Table S6). Bioinformatics analysis of the *Thbs1* promoter region further identified three potential Stat3 binding sites (Figure S17b,c), indicating that *Thbs1* may be a direct Stat3 target. Consistent with this prediction, treatment of BMSCs with M1‐like STIMs CM enhanced Stat3 phosphorylation and increased Thbs1 expression (Figure S17d). ELISA assays further showed that Thbs1 concentration was higher in CM collected from M1‐like STIMs CM‐treated BMSCs than in CM collected from M0 CM‐treated BMSCs (29.11 ± 1.02 vs. 15.06 ± 0.44 ng/mL; Figure S17e). Conversely, Stat3 knockdown in BMSCs reduced *Thbs1* mRNA and protein expression (Figure S17f–h). ChIP analysis further demonstrated that Stat3 specifically enriched the *Thbs1* promoter fragment containing predicted binding site 3, suggesting that Stat3 functions as a key transcriptional activator of *Thbs1* (Figure S17i). Collectively, these findings support a self‐amplifying pro‐inflammatory feedback loop in which senescent BMSC‐derived Thbs1 drives macrophage M1 polarization and IL‐6 secretion. IL‐6 subsequently activates JAK/STAT3 signaling in BMSCs, and activated Stat3 potentially upregulates *Thbs1* transcription, sustaining chronic inflammation and osteogenic suppression.

### 3.9 AAV9‐Mediated Thbs1 Knockdown Reprograms Macrophages and Improves Age‐Related Bone Repair

To determine whether senescent BMSC‐derived Thbs1 impairs bone regeneration in vivo by suppressing macrophage mitophagy, we employed a naturally aged rat critical‐sized calvarial defect model and locally delivered an AAV9 vector encoding a Thbs1‐targeting shRNA (AAV9‐sh‐Thbs1‐mScarlet). AAV9 has been reported to transduce BMSCs under defined experimental conditions (Yang et al. 2022; Lin et al. 2024). Four weeks after local administration, cells isolated from the defect‐region calvarial bone were first characterized by flow cytometry and exhibited a CD90^+^CD105^+^CD34^−^CD45^−^ phenotype, consistent with BSMC characteristics (Figure S18a). In these defect‐region BMSCs, local AAV‐mediated transduction and Thbs1 knockdown were verified by robust mScarlet fluorescence and significant reduction of Thbs1 protein in the AAV9‐sh‐Thbs1 group compared with the AAV9‐sh‐NC group (Figure S18b,c).

**FIGURE 6:**
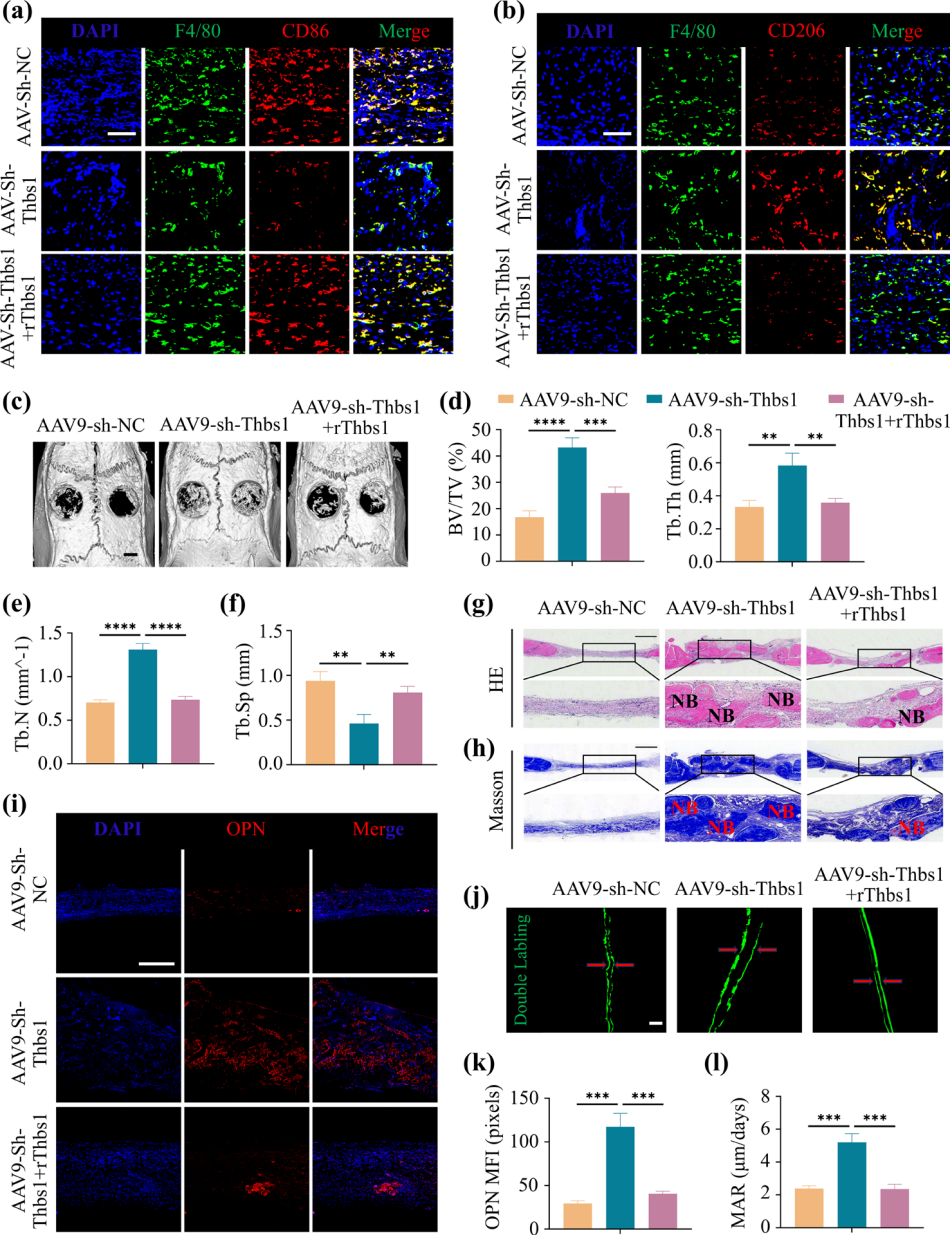
AAV9‐mediated knockdown of BMSC‐derived Thbs1 reprograms macrophages to improve bone regeneration in aged rats. Naturally aged rats were sacrificed 1 month after calvarial defect surgery (*n* = 12 per group). (a, b) Representative IF images of F4/80^+^CD86^+^ (M1) and F4/80^+^CD206^+^ (M2) macrophages in calvarial bone defects treated with AAV9‐sh‐NC, AAV9‐sh‐Thbs1, or AAV9‐sh‐Thbs1 + rThbs1 (*n* = 6). Scale bar: 25 μm. (c) Representative micro‐CT images of calvarial bone defects (*n* = 12). Scale bar: 2 mm. (d–f) Quantitative analysis of bone parameters, including BV/TV, Tb.Th, Tb.N, and Tb.Sp (*n* = 6). (g, h) Histological evaluation of defect regions by H&E staining (g) and Masson's trichrome staining (h) (NB, new bone) (*n* = 8). Scale bar: 50 μm. (i) IF images of osteogenic marker OPN (red) with DAPI (blue) (*n* = 6). Scale bar: 25 μm. (j) Representative double‐labeling images revealing mineralizing surfaces (red lines) (*n* = 5). Scale bar: 25 μm. (k) Quantification of osteopontin (OPN) MFI (*n* = 6). (l) Quantification of mineral apposition rate (*n* = 5). Data are presented as mean ± SD. Statistical significance (**p < 0.01; ***p < 0.001; ****p < 0.0001) was assessed using one‐way ANOVA with Šídák's multiple comparisons test (d, e, f, k, l).

Comprehensive analysis revealed that macrophages isolated from Thbs1‐knockdown defects exhibited reduced total ROS, mitochondrial superoxide levels, and restored MMP (Figure S19a–c), indicating a marked improvement in mitochondrial function. IF staining of tissue sections further demonstrated that Thbs1 knockdown alleviated mitophagy inhibition, as reflected by increased TOMM20–LC3B colocalization (Figure S19d), and was accompanied by an elevated proportion of CD206^+^ M2 macrophages (Figure 6a,b). Consistently, flow cytometric analysis revealed a decreased proportion of M1 macrophages (CD86^+^) and an increase in M2 macrophages (CD206^+^) in the AAV9‐sh‐Thbs1‐treated group (Figure S19e,f). Notably, these benefits were reversed by exogenous rThbs1 (Figure S19a–f and Figure 6a,b), highlighting the specificity of Thbs1 as an upstream driver.

Having established that Thbs1 targeting reprograms macrophage function, we assessed its impact on bone repair in the aged skeleton. AAV9‐sh‐Thbs1 treatment significantly enhanced new bone formation compared with empty‐vector controls, as evidenced by increased BV/TV, Tb.Th, and Tb.N, and reduced Tb.Sp (Figure 6c–f). H&E and Masson's trichrome staining further demonstrated more extensive new bone formation and denser collagen deposition within the defect region (Figure 6g,h). Consistently, OPN IF was markedly enhanced in the AAV9‐sh‐Thbs1 group (Figure 6i,k), and calcein double labeling confirmed a higher mineral apposition rate (Figure 6j,l). Notably, these pro‐regenerative effects were partially reversed by rThbs1 add‐back (Figure 6c–l). Collectively, these in vivo results demonstrate that AAV9‐mediated Thbs1 knockdown alleviates age‐related bone repair failure by restoring macrophage mitophagy, improving mitochondrial function, and correcting M1/M2 polarization imbalance, thereby providing strong preclinical support for Thbs1 as a therapeutic target for aging‐related bone defects.

## 4 Discussion

Marrow inflammaging and redox homeostasis disruption are pivotal drivers of the age‐related decline in bone regenerative capacity (Kushioka et al. 2023; Stegen et al. 2016). This pathological state is primarily characterized by the abnormal accumulation of senescent BMSCs and M1‐polarized macrophages (Li et al. 2023). However, the core signaling molecules mediating stromal–immune crosstalk that sustain this pathological loop remain poorly defined. Here, we identify Thbs1 as a prominent BMSC‐derived SASP component that promotes macrophage M1 polarization, thereby exacerbating age‐related bone regeneration failure. Mechanistically, Thbs1 binds to Tgfbr2 and activates Smad3 signaling to transcriptionally repress *Pink1* expression, impairing PINK1/Parkin‐mediated mitophagy and inducing macrophage mitochondrial dysfunction. Furthermore, we uncover a reciprocal feedback loop in which IL‐6 from Thbs1‐activated M1 macrophages activates JAK/STAT3 signaling in BMSCs, inhibiting osteogenic differentiation while simultaneously upregulating *Thbs1* transcription. Together, these findings establish Thbs1 as a central regulator of marrow inflammaging and a potential therapeutic target for restoring BMM homeostasis and enhancing aging‐related bone regeneration.

Beyond their role as osteoprogenitors, BMSCs are key regulators of bone immune balance (Zhang et al. 2025). This immunoregulatory function is largely mediated by paracrine signaling, involving the local release of bioactive cytokines and growth factors that shape inflammatory and repair responses. For example, BMSC‐derived soluble factors such as vascular endothelial growth factor (VEGF), interleukin‐10 (IL‐10), and transforming growth factor‐beta 1 (TGF‐β1) can modulate inflammation and support a pro‐regenerative niche (Abdelmohsen et al. 2026). However, aging fundamentally reprograms BMSC function, compromising their immunoregulatory competence and diverting their secretome to drive pro‐inflammatory M1 macrophage polarization (Pajarinen et al. 2019; Yin et al. 2017). This disruption is conventionally attributed to the canonical SASP, which is enriched in pleiotropic cytokines such as IL‐6, IL‐1β, and TNF‐α (Massaro et al. 2023; Li et al. 2025; Zhang et al. 2024). However, targeting these downstream soluble mediators is often limited by redundancy and compensatory signaling. The upstream niche‐remodeling matricellular signals that may initiate and sustain maladaptive stromal–immune crosstalk remain insufficiently elucidated. In this study, we identify Thbs1 as not only a dominant SASP factor produced by senescent BMSCs, but more importantly, as a key upstream driver within this secretory program. As a matricellular signal, Thbs1 remodels the extracellular niche and drives macrophages toward an M1‐like phenotype. These findings position Thbs1 as a critical senescence‐induced matricellular mediator that links stromal senescence to macrophage inflammatory reprogramming in the aging BMM. Notably, this concept aligns with recent observations in renal aging, where Thbs1 promotes macrophage inflammatory transformation (Kang et al. 2025), suggesting that Thbs1‐driven immune reprogramming may represent a conserved feature of tissue aging.

Sustained M1‐like macrophage polarization is a central mediator of age‐related bone regeneration failure, perpetuating chronic inflammation and impairing tissue repair (Li et al. 2021). Mitochondrial dysfunction is a key mechanism driving this pro‐inflammatory shift in macrophages (Chen et al. 2025). Mitochondria are a major source of ROS, and MMP is essential for maintaining cellular energy metabolism and functional integrity (Hu et al. 2024). Previous research establishes that electron transport chain disruption and mitochondrial membrane depolarization can cause excessive ROS accumulation, thereby promoting M1 polarization and amplifying inflammatory cascades (Wang et al. 2025). Mitophagy, a protective mechanism, selectively degrades damaged mitochondria to maintain MQC and redox homeostasis (Xu et al. 2025; Yang et al. 2024). Recent research indicates that defective mitophagy exacerbates mitochondrial dysfunction and is closely linked to the progression of inflammatory diseases. For instance, Meng et al. (2021) reported that mitophagy is impaired in inflammation‐stimulated macrophages, correlating with M1 polarization. Chen et al. (2025) demonstrated that restoring mitophagy suppresses M1 macrophage polarization and attenuates periodontitis‐induced bone loss. However, the upstream signaling mechanisms that disrupt MQC within the aging BMM remain elusive.

Thbs1, a multifunctional matricellular protein, plays a critical role in immune regulation and tissue homeostasis (Ramalingam et al. 2025). However, previous studies primarily focus on correlations between Thbs1 and inflammatory phenotypes (Hassan et al. 2024; Liang and Zhang 2025), and a mechanistic understanding of how Thbs1 regulates mitochondrial homeostasis to dictate macrophage fate is still lacking. Here, we demonstrate that Thbs1 suppresses mitophagy, leading to damaged mitochondria accumulation, MMP loss, and elevated total and mitochondrial ROS, ultimately promoting an M1‐like phenotype. Previous work in non‐skeletal tissues supports a direct impact of THBS1 on mitochondrial homeostasis. For instance, in a viral infection model, THBS1 upregulation increases mitochondrial Ca^2+^ levels and decreases MMP (Zhao et al. 2019). Moreover, treatment with Thbs1 or its CD47‐binding domain induces MMP loss and ROS accumulation (Roberts and Isenberg 2021). Extending these observations of Thbs1‐driven mitochondrial dysfunction, our findings position mitophagy as a key mechanism through which Thbs1 regulates macrophage mitochondrial homeostasis and inflammatory phenotypes. To our knowledge, this is the first study to report that Thbs1 modulates macrophage polarization via mitophagy during aging.

PINK1/Parkin‐mediated mitophagy is a key MQC pathway that maintains mitochondrial function and restrains oxidative stress (Wang et al. 2025; Narendra et al. 2010). In the aging BMM, we demonstrate that Thbs1 activates TGF‐β/Smad3 signaling in macrophages and represses *Pink1* transcription by promoting Smad3 nuclear translocation, thereby selectively suppressing PINK1/Parkin‐mediated mitophagy. This contrasts with a recent report in HeLa cells, where SMAD3 transcriptionally enhanced *PINK1* expression (Tang et al. 2025). This discrepancy likely reflects major differences in cell type and stress context. While the earlier study examined an epithelial cancer cell line exposed to acute mitochondrial damage, we focused on naturally aged rat BMDMs in a chronically inflamed BMM. These findings highlight the strong context dependence of Smad3 transcriptional output. One possible explanation is that aging‐associated chronic inflammation reprograms Smad3 transcriptional function. Unlike the transient mitochondrial stress observed in HeLa cells, sustained inflammation in aging BMM may induce specific post‐translational modifications (Zhao et al. 2018; Zhou et al. 2022) or recruit specific co‐repressors (Chen et al. 2002), shifting Smad3 toward a transcriptionally repressive role. Therefore, elucidating how cellular context and stress duration shape Smad3 transcriptional output will be an important direction for future research.

Macrophages are central effectors of innate immunity and play a pivotal role in the pathogenesis of age‐related impairment in bone regeneration. Li et al. (2021) demonstrated that grancalcin (GCA) released by senescent immune cells inhibits osteogenesis in BMSCs, thereby accelerating skeletal aging. More recently, Zou et al. (2024) extended this concept to fracture repair, showing that GCA from callus macrophages induces secondary senescence and mitochondrial dysfunction in skeletal progenitors and delays fracture healing in aged mice. These studies underscore pathogenic stromal–immune crosstalk as a major driver of impaired bone repair with aging. However, prior work has largely framed stromal–immune interactions in aging as unidirectional, emphasizing either immune‐to‐stromal regulation or stromal‐driven remodeling of the immune landscape. Here, we uncover a self‐amplifying positive feedback loop that transforms this crosstalk into a fully bidirectional regulatory program during skeletal aging. Senescent BMSCs, through Thbs1, induce a senescence‐like state in macrophages, polarizing them into a distinct population of M1‐like STIMs. These M1‐like STIMs, in turn, secrete factors that inhibit BMSC osteogenic capacity, thereby completing the feedback loop.

We further identify IL‐6 as a key effector within this loop. Although IL‐6 is well established to inhibit osteogenesis and promote osteoclastogenesis (Tanaka et al. 2014; Hou and Tian 2022), our study reveals a specific pathogenic context in aging. We demonstrated that M1‐like STIM–derived IL‐6 feeds back on senescent BMSCs—which exhibit heightened sensitivity to IL‐6 signaling—to inhibit osteogenic differentiation via JAK/STAT3 activation, consistent with established mechanisms of IL‐6‐mediated bone impairment (Hou and Tian 2022; Sims et al. 2004). Notably, activated Stat3 binds the *Thbs1* promoter and transcriptionally enhances its expression, thereby sustaining macrophage senescence‐like state and inflammatory output. To our knowledge, this work delineates a previously unrecognized Thbs1–STIMs–IL‐6/STAT3 positive feedback loop that perpetuates pathological stromal–immune crosstalk in the aging BMM, providing a mechanistic explanation for the persistence of inflammaging and the progressive failure of bone regeneration.

We demonstrated that a Thbs1‐driven stromal–immune feedback loop is a key mechanism underlying the disturbed BMM during aging. However, the sex‐specific relevance of this regulatory axis remains to be clarified. Sex differences in bone health and healing are well documented, and osteoporosis is particularly prevalent in postmenopausal females, in whom estrogen deficiency represents a major pathogenic driver of skeletal deterioration and impaired bone homeostasis (Ortona et al. 2023; Qi et al. 2017). The present study was performed exclusively in aged male rats. Thus, our findings should be interpreted primarily in the context of male skeletal aging and should not be directly extrapolated to female skeletal aging, particularly postmenopausal skeletal deterioration and regenerative impairment. This sex bias may limit the generalizability of our conclusions and could have implications for clinical translation, as therapeutic strategies targeting the Thbs1‐driven feedback loop may exhibit sex‐dependent efficacy. Further studies using aged female animals and postmenopausal osteoporosis models are warranted to determine whether this Thbs1‐centered mechanism is conserved in female skeletal aging. Addressing this issue will be essential for defining the broader biological relevance and translational potential of this regulatory axis in age‐related skeletal disorders across both sexes.

Despite uncovering a new regulatory network, there are limitations in this study. Although we demonstrated Smad3 binding to the *Pink1* promoter, the precise mechanism by which transcriptional repression occurs remains unclear. Specific co‐repressors or epigenetic modifications may be required. Similarly, while our work identified Tgfbr2 as a critical receptor for Thbs1, we cannot exclude contributions from other known Thbs1 receptors, such as CD36 or CD47, in regulating macrophage mitophagy and polarization. Addressing these questions in future studies will further clarify the broader role of this Thbs1‐centered axis in age‐related diseases.

In summary, this study elucidates a pathogenic mechanism in which aging BMSCs suppress macrophage mitophagy and drive M1 polarization via a Thbs1/TGF‐β/Smad3/PINK1 axis, while a reciprocal IL‐6/JAK/STAT3 feedback loop sustains chronic inflammation and impairs bone repair. Targeting Thbs1 to restore MQC and resolve inflammaging may represent a promising therapeutic strategy, not only for age‐related skeletal decline but also for multiple age‐related diseases and tissue regeneration.

## Author Contributions

**Yifeng Xing and Jingjing Su:** writing – review and editing, writing – original draft, visualization, validation, project administration, methodology, conceptualization, investigation, formal analysis, data curation. **Yanjun Lin and Nengwen Huang:** writing – original draft, visualization, validation, methodology, formal analysis. **Sihui Zhang, Yuwei Zhou, and Jie Lu:** validation, software, methodology. **Weiping Chen, Kaixun He, and Wenxiu Yuan:** visualization, validation, methodology, investigation. **Yang Li, Geyuan Zheng, and Pengyuan Hu:** methodology, investigation, formal analysis, software. **Dong Wu and Yanjing Ou:** writing – review and editing, visualization, validation, project administration, supervision, conceptualization. **Jiang Chen:** writing – review and editing, resources, validation, supervision, project administration, methodology, funding acquisition, conceptualization.

## Funding

This work was supported by the National Natural Science Foundation of China (Nos 82371008 and 81771126).

## Conflicts of Interest

The authors declare no conflicts of interest.

## Supporting information


**Table S1:** ChIP‐PCR primer sequences.
**Table S2:** AAV‐shRNA construct sequences.
**Table S3:** Venn diagram of the overlap among differentially expressed genes in extracellular matrix (EMEGs), immune response (IREGs), and senescence‐associated (SAEGs).
**Table S4:** Differential remodeling of the JAK–STAT pathway in aged BMSCs.
**Table S5:** GO terms and associated genes shared across aging‐related processes.
**Table S6:** Predicted Stat3‐binding sites in gene promoters.

**Figure S1:** Phenotypic characterization and senescence‐related associated marker profiles of young and aged BMSCs and BMDMs.
**Figure S2:** Aging promotes a pro‐inflammatory M1‐like phenotype in BMDMs.
**Figure S3:** Senescent BMSC secretome impairs mitochondrial function and mitophagy in macrophages.
**Figure S4:** Thbs1 is upregulated in aged BMSCs.
**Figure S5:** Efficient knockdown of Thbs1 in BMSCs.
**Figure S6:** Thbs1‐neutralizing antibody treatment suppresses macrophage M1 polarization.
**Figure S7:** Thbs1 impairs BMDMs' mitochondrial function and mitophagy.
**Figure S8:** Thbs1 activates TGF‐β/Smad3 signaling by binding to the Tgfbr2 on macrophages.
**Figure S9:** Thbs1 promotes macrophage M1 polarization via TGF‐β/Smad3 signaling.
**Figure S10:** Pharmacological inhibition of Smad3 partially reverses Thbs1‐driven M1 polarization in BMDMs.
**Figure S11:** Thbs1 activates TGF‐β/Smad3 signaling to disrupt mitochondrial redox balance and membrane potential in BMDMs.
**Figure S12:** Thbs1 activates TGF‐β/Smad3 signaling to suppress mitophagy in BMDMs through TGF‐β/Smad3 signaling.
**Figure S13:** Thbs1 impairs mitochondrial redox balance and mitophagy in BMDMs via Smad3 activation.
**Figure S14:** Impaired cranial bone regeneration in aged rats is correlated with enhanced M1 macrophage polarization.
**Figure S15:** Thbs1 secreted by aged BMSCs suppresses osteogenesis through an M1 macrophage‐mediated feedback loop.
**Figure S16:** IL‐6 neutralization rescues M1‐like‐STIM‐mediated inhibition of osteogenesis.
**Figure S17:** Stat3 transcriptionally activates *Thbs1* to sustain a pro‐inflammatory feedback loop in aged BMSCs.
**Figure S18:** AAV9‐sh‐Thbs1‐mScarlet efficiently silences Thbs1 in BMSCs in vivo.
**Figure S19:** AAV9‐mediated knockdown of BMSC‐derived Thbs1 restores macrophage mitochondrial function, mitophagy, and polarization in aged rat calvarial defects.

## Data Availability

The data that supports the findings of this study are available in the Supporting Material of this article.
