# Supplementary material for: Senescent BMSC‐Derived Thbs1 Drives Inflammaging and Impairs Bone Regeneration by Suppressing PINK1/Parkin‐Mediated Mitophagy in Macrophages
Source: Aging Cell. 2026 Jun 8;25(6):e70575. doi: 10.1111/acel.70575 (PMC13244667; doi:10.1111/acel.70575)
Supplement: Supplementary file 2 — Figure S1: Phenotypic characterization and senescence‐related associated marker profiles of young and aged BMSCs and BMDMs. Figure S2: Aging promotes a pro‐inflammatory M1‐like phenotype in BMDMs. Figure S3: Senescent BMSC secretome impairs mitochondrial function and mitophagy in macrophages. Figure S4: Thbs1 is upregulated in aged BMSCs. Figure S5: Efficient knockdown of Thbs1 in BMSCs. Figure S6: Thbs1‐neutralizing antibody treatment suppresses macrophage M1 polarization. Figure S7: Thbs1 impairs BMDMs' mitochondrial function and mitophagy. Figure S8: Thbs1 activates TGF‐β/Smad3 signaling by binding to the Tgfbr2 on macrophages. Figure S9: Thbs1 promotes macrophage M1 polarization via TGF‐β/Smad3 signaling. Figure S10: Pharmacological inhibition of Smad3 partially reverses Thbs1‐driven M1 polarization in BMDMs. Figure S11: Thbs1 activates TGF‐β/Smad3 signaling to disrupt mitochondrial redox balance and membrane potential in BMDMs. Figure S12: Thbs1 activates TGF‐β/Smad3 signaling to suppress mitophagy in BMDMs through TGF‐β/Smad3 signaling. Figure S13: Thbs1 impairs mitochondrial redox balance and mitophagy in BMDMs via Smad3 activation. Figure S14: Impaired cranial bone regeneration in aged rats is correlated with enhanced M1 macrophage polarization. Figure S15: Thbs1 secreted by aged BMSCs suppresses osteogenesis through an M1 macrophage‐mediated feedback loop. Figure S16: IL‐6 neutralization rescues M1‐like‐STIM‐mediated inhibition of osteogenesis. Figure S17: Stat3 transcriptionally activates Thbs1 to sustain a pro‐inflammatory feedback loop in aged BMSCs. Figure S18: AAV9‐sh‐Thbs1‐mScarlet efficiently silences Thbs1 in BMSCs in vivo. Figure S19: AAV9‐mediated knockdown of BMSC‐derived Thbs1 restores macrophage mitochondrial function, mitophagy, and polarization in aged rat calvarial defects. [file ACEL-25-e70575-s002.docx]

**Supplementary figures**

**Senescent BMSC-derived Thbs1 drives inflammaging and impairs bone regeneration by suppressing PINK1/Parkin-mediated mitophagy in macrophages**

Yifeng Xing ^1,2,a^, Jingjing Su ^3,a^, Yanjun Lin ^1^, Nengwen Huang ^1,2^, Sihui Zhang ^1^, Yuwei Zhou ^4^, Jie Lu ^1,2^, Weiping Chen ^1,2^, Kaixun He ^1^, Wenxiu Yuan ^1^, Yang Li ^5^, Geyuan Zheng ^1,2^, Pengyuan Hu ^1,2^, Dong Wu ^1^, Yanjing Ou ^1^, Jiang Chen ^1,2^

**Contents:**

**FIGURE S1. Phenotypic characterization and senescence-related associated marker profiles of young and aged BMSCs and BMDMs.**

**FIGURE S2. Aging promotes a pro‑inflammatory M1-like phenotype in BMDMs.**

**FIGURE S3. Senescent BMSC secretome impairs mitochondrial function and mitophagy in macrophages.**

**FIGURE S4. Thbs1 is upregulated in aged BMSCs.**

**FIGURE S5. Efficient knockdown of Thbs1 in BMSCs.**

**FIGURE S6. Thbs1-neutralizing antibody treatment suppresses macrophage M1 polarization.**

**FIGURE S7. Thbs1 impairs BMDMs' mitochondrial function and mitophagy.**

**FIGURE S8. Thbs1 activates TGF-β/Smad3 signaling by binding to the Tgfbr2 on macrophages.**

**FIGURE S9. Thbs1 promotes macrophage M1 polarization via TGF-β/Smad3 signaling.**

**FIGURE S10. Pharmacological inhibition of Smad3 partially reverses Thbs1‑driven M1 polarization in BMDMs.**

**FIGURE S11. Thbs1 activates TGF-β/Smad3 signaling to disrupt mitochondrial redox balance and membrane potential in BMDMs.**

**FIGURE S12. Thbs1 activates TGF-β/Smad3 signaling to suppress mitophagy in BMDMs through TGF‑β/Smad3 signaling.**

**FIGURE S13. Thbs1 impairs mitochondrial redox balance and mitophagy in BMDMs via Smad3 activation.**

**FIGURE S14. Impaired cranial bone regeneration in aged rats is correlated with enhanced M1 macrophage polarization.**

**FIGURE S15. Thbs1 secreted by aged BMSCs suppresses osteogenesis through an M1 macrophage‑mediated feedback loop.**

**FIGURE S16. IL‑6 neutralization rescues M1‑like‑STIM‑mediated inhibition of osteogenesis.**

**FIGURE S17. Stat3 transcriptionally activates *Thbs1* to sustain a pro‑inflammatory feedback loop in aged BMSCs.**

**FIGURE S18. AAV9‑sh‑Thbs1‑mScarlet efficiently silences Thbs1 in BMSCs in vivo.**

**FIGURE S19. AAV9‑mediated knockdown of BMSC‑derived Thbs1 restores macrophage mitochondrial function, mitophagy, and polarization in aged rat calvarial defects.**


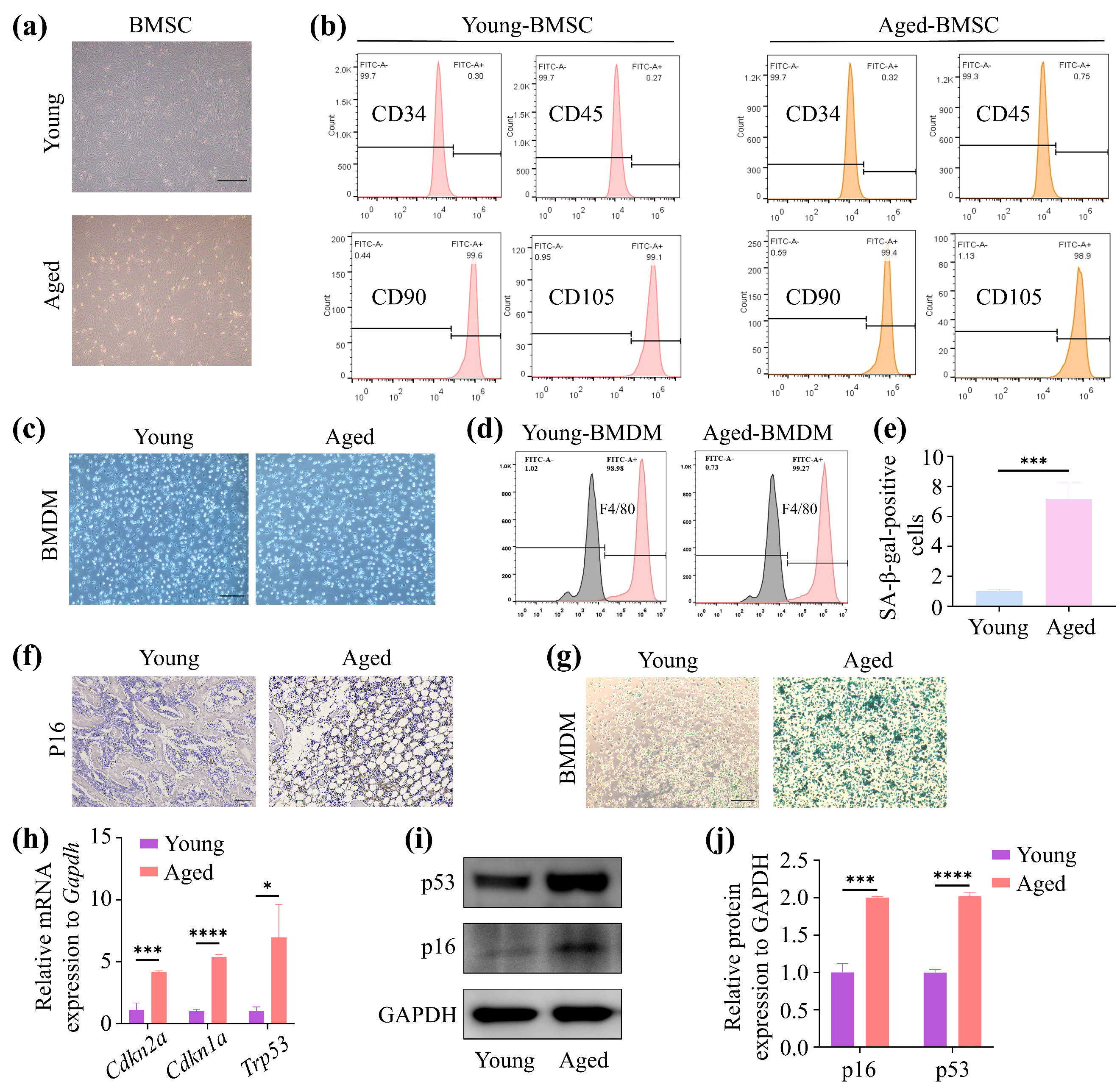


**FIGURE S1. Phenotypic characterization and senescence-associated marker profiles of young and aged BMSCs and BMDMs.**

**(a)** Representative morphology of young and aged BMSCs (passage 3; Scale bar:  100 μm) (n = 3). **(b)** Flow cytometric analysis of BMSC surface markers (CD34, CD45, CD90, and CD105) (n = 3). **(c)** Representative morphology of young and aged BMDMs (n = 3). Scale bar: 100 μm. **(d)** Flow cytometric analysis of the macrophage marker F4/80 in BMDMs (n = 3). **(e)** Quantification of SA-β-gal staining in BMSCs (n = 3). **(f)** Representative immunohistochemical staining of p16 in femoral sections from young and aged rats (n = 3). Scale bar: 100 μm. **(g)** Representative SA-β-gal staining of young and aged BMDMs (n = 3). Scale bar: 100 μm. **(h)** mRNA expression levels of senescence-associated genes (*Cdkn2a*, *Cdkn1a*, and *Trp53*) in BMDMs (n = 3). **(i, j)** Western blot analysis **(i)** and quantification **(j)** of p53 and p16 protein expression in BMDMs (n = 3). Data are presented as mean  ±  SD. Statistical significance (**p* < 0.05; ****p* < 0.001; *****p* < 0.0001) was assessed using unpaired two-tailed Student's *t*-test (e, h, and j).


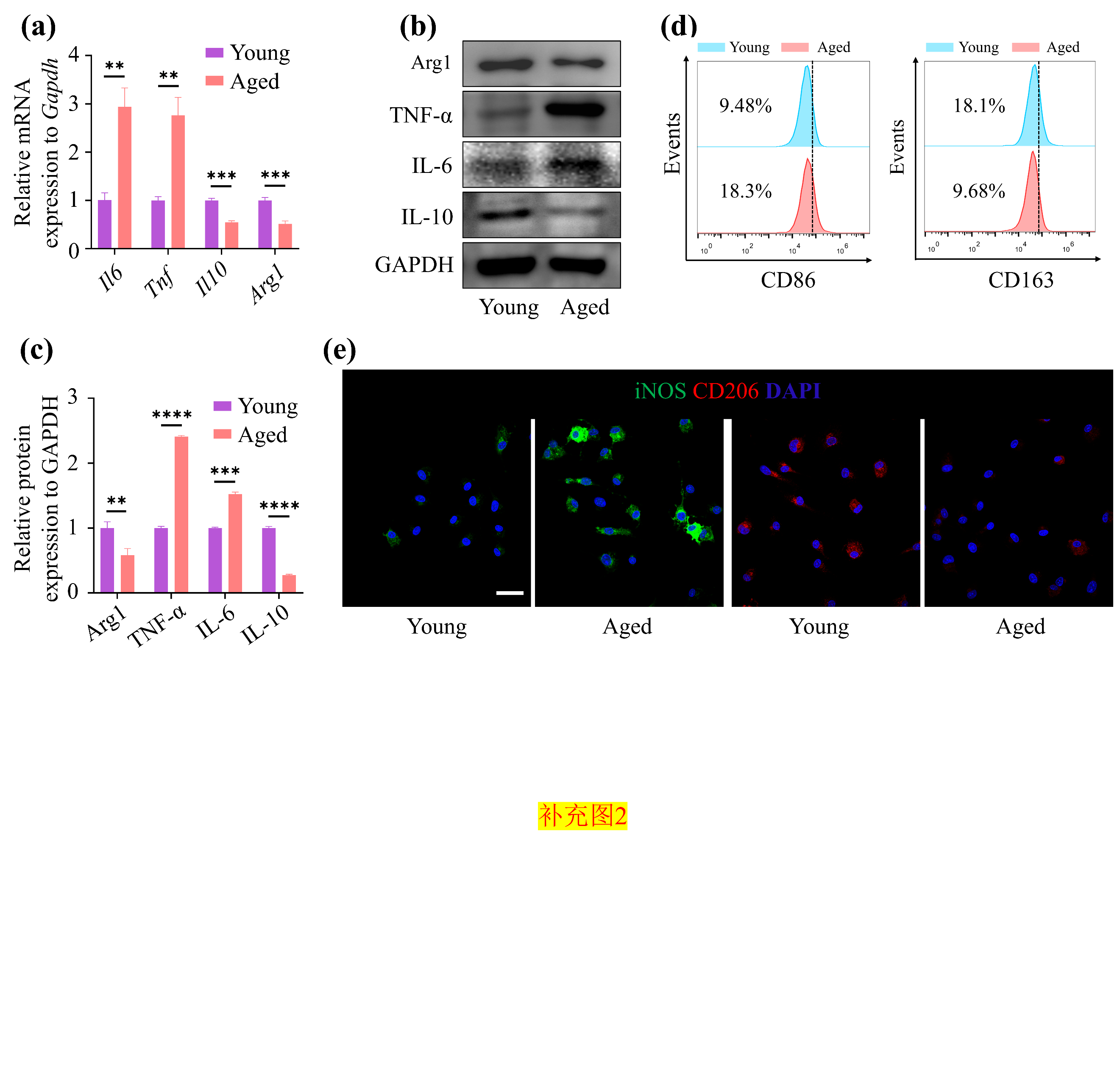


**FIGURE S2. Aging promotes a pro‑inflammatory M1-like phenotype in BMDMs.**

**(a)** mRNA expression levels of M1-associated (*Il6* and *Tnf*) and M2-associated (*Il10* and *Arg1*) genes in young and aged BMDMs (n = 3). **(b, c)** Western blot analysis **(b)** and quantification **(c)** of Arg1, TNF-α, IL-6, and IL-10 protein expression in BMDMs (n = 3). **(d)** Flow cytometry analysis of M1 (CD86) and M2 (CD163) surface marker expression in BMDMs (n = 3). **(e)** IF staining of M1 (iNOS) and M2 (CD206) markers in young and aged BMDMs (n = 4). Scale bar: 25 μm. Data are presented as mean  ±  SD. Statistical significance (***p* < 0.01; ****p* < 0.001; *****p* < 0.0001) was assessed using unpaired two-tailed Student's *t*-test (a and c).


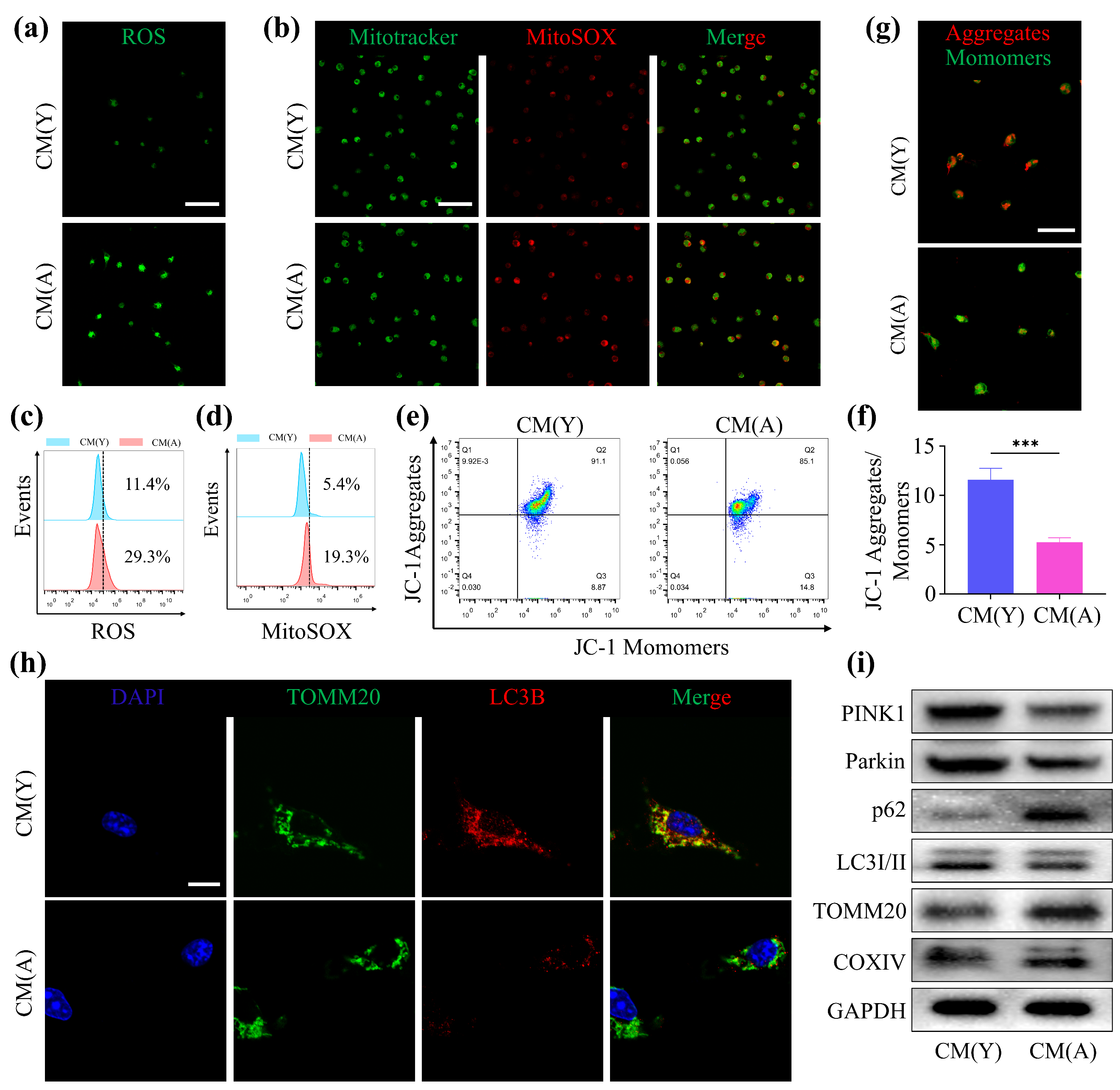


**FIGURE S3. Senescent BMSC secretome impairs mitochondrial function and mitophagy in macrophages.**

BMDMs were treated for 48 h with CM(Y) or CM(A). **(a)** Representative fluorescence images of DCF-DA staining (green) revealing total intracellular ROS in BMDMs (n = 3). Scale bar: 50 μm. **(b)** Mitochondrial ROS detection in BMDMs by MitoTracker (green) and MitoSOX (red) staining (n = 3). Scale bar: 50 μm. **(c)** Flow cytometric analysis of total ROS levels in BMDMs (n = 3). **(d)** Flow cytometric analysis of mitochondrial ROS levels in BMDMs stained with MitoSOX Red (n = 3). **(e, f)** Flow cytometric assessment of mitochondrial membrane potential (aggregate‑to‑monomer ratio) in BMDMs (n = 3). Data are presented as mean  ±  SD. Statistical significance (****p* < 0.001) was assessed using unpaired two-tailed Student's *t*-test (f). **(g)** IF analysis of JC-1 aggregates (red) and monomers (green) in BMDMs (n = 3). Scale bar: 50 μm. **(h)** Representative fluorescence images with TOMM20 (green) and LC3B (red) double-staining in BMDMs (n = 3). Scale bar: 10 μm. **(i)** Western blot analysis of PINK1, Parkin, p62, LC3I/II, TOMM20, and COXIV expression levels in BMDMs (n = 3).


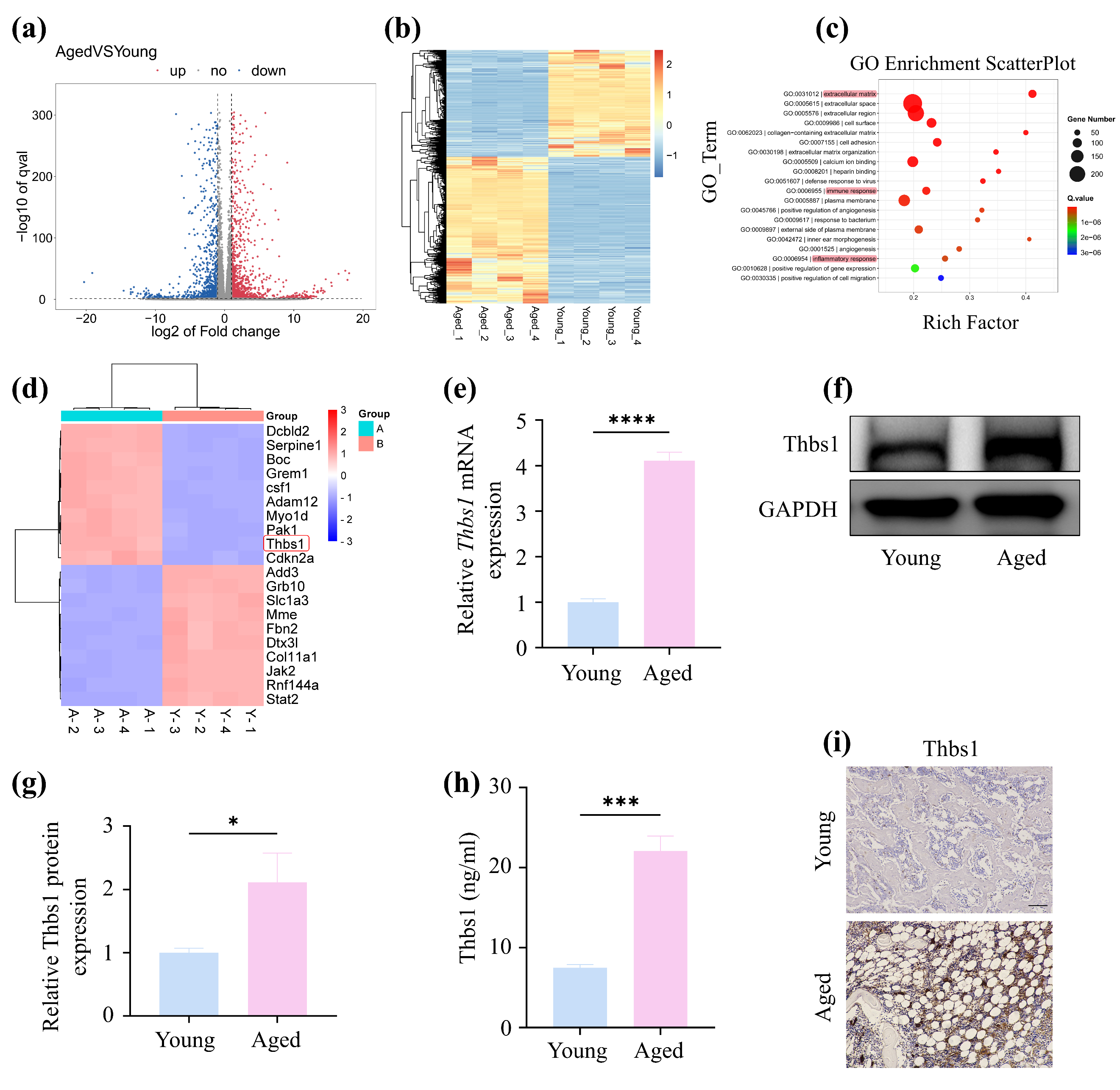


**FIGURE S4. Thbs1 is upregulated in aged BMSCs.**

BMSCs were isolated from young and aged rats and cultured to passage 3 for analysis. **(a)** Volcano plot of RNA-sequencing differentially expressed genes (DEGs) (n = 4). **(b)** Heatmap of RNA-sequencing transcriptomic profiles in young and aged BMSCs (n = 4). **(c)** Gene ontology (GO) analysis of biological processes enriched among DEGs in aged BMSCs (n = 4). **(d)** Heatmap of DEGs (fold change ≥ 2, *p* ≤ 0.05) (n = 4). **(e)** mRNA expression levels of Thbs1 in young and aged BMSCs (n = 3). **(f, g)** Western blot analysis **(f)** and quantification **(g)** of Thbs1 protein expression in BMSCs (n = 3). **(h)** ELISA quantification of Thbs1 in CM from young and aged BMSCs (n = 3). **(i)** Representative immunohistochemical staining of Thbs1 in femoral sections from young and aged rats (n = 5). Scale bar: 100 μm. Data are presented as mean  ±  SD. Statistical significance (**p* < 0.05; ****p* < 0.001; *****p* < 0.0001) was assessed using unpaired two-tailed Student's *t*-test (e, g, and h).


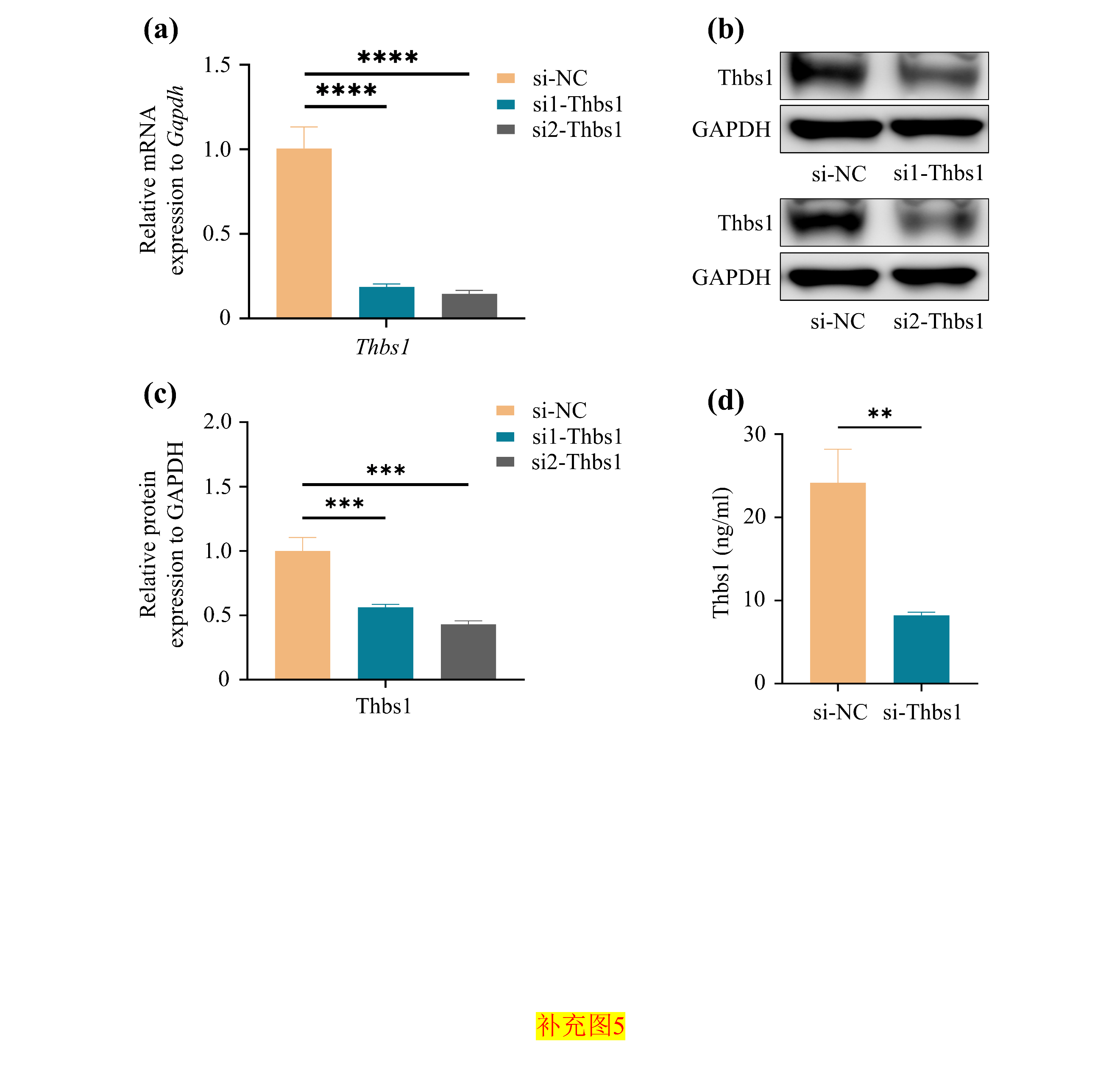


**FIGURE S5. Efficient knockdown of Thbs1 in BMSCs.**

BMSCs were transfected with two independent siRNAs targeting Thbs1 (si1‑Thbs1 and si2‑Thbs1) or a non‑targeting control siRNA (si‑NC). **(a)** mRNA expression levels of Thbs1 (n = 3). **(b, c)** Western blot analysis **(b)** and quantification **(c)** of Thbs1 protein expression (n = 3). **(d)** ELISA quantification of Thbs1 in CM from transfected BMSCs (n = 3). Data are presented as the mean ± SD. Statistical significance (***p* < 0.01; ****p* < 0.001; *****p* < 0.0001) was assessed using unpaired two-tailed Student's *t*-test (d) or one-way ANOVA with Dunnett's test (a and c).


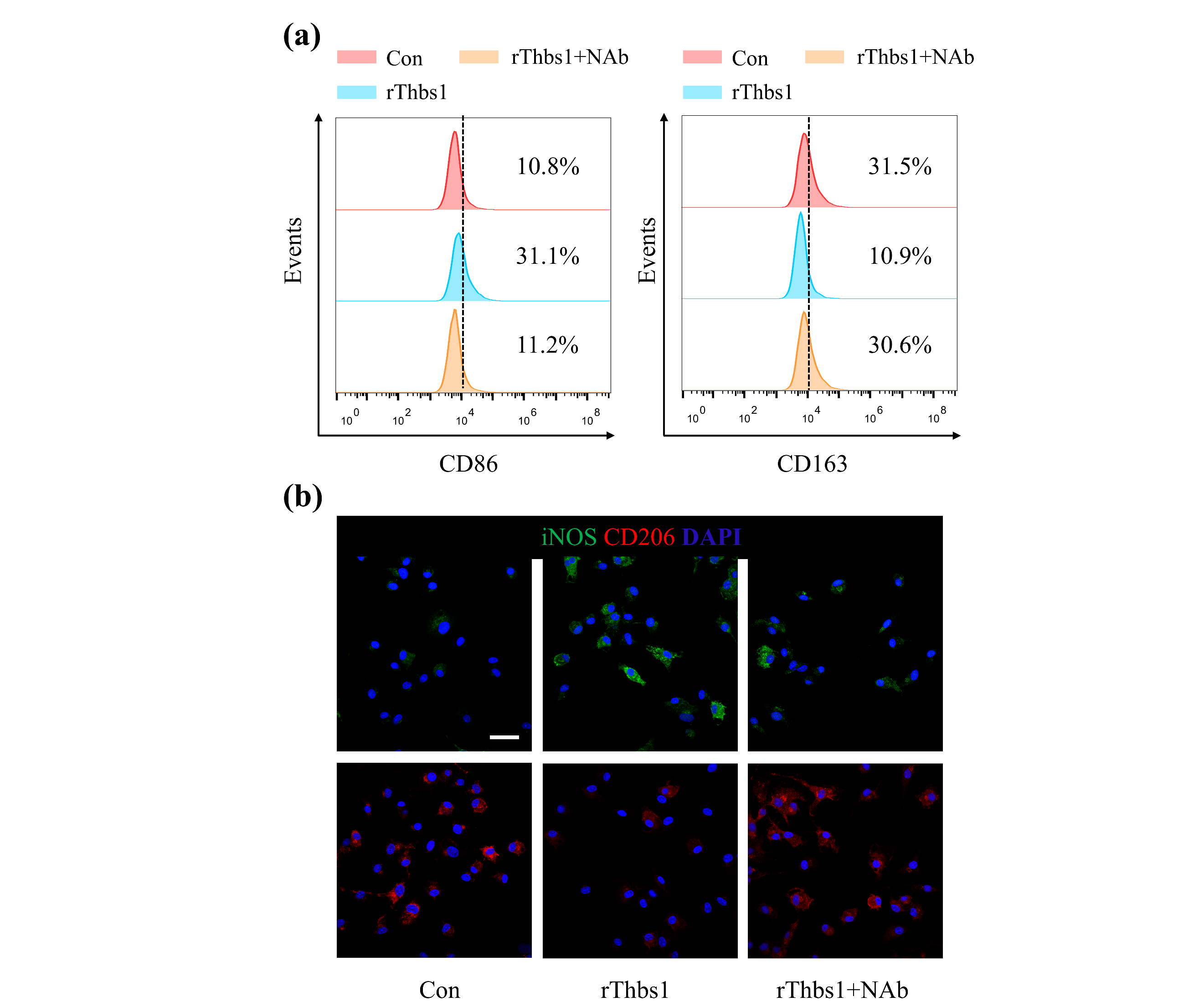


**FIGURE S6.** **Thbs1-NAb treatment suppresses macrophage M1 polarization.**

**(a)** Flow cytometric analysis of M1 (CD86) and M2 (CD163) surface markers on BMDMs treated with Thbs1 and in addition with or without Thbs1-NAb treatment (n = 3). **(b)** IF of M1 (iNOS) and M2 (CD206) markers in BMDMs treated as in (a) (n = 4). Scale bar: 25 μm.


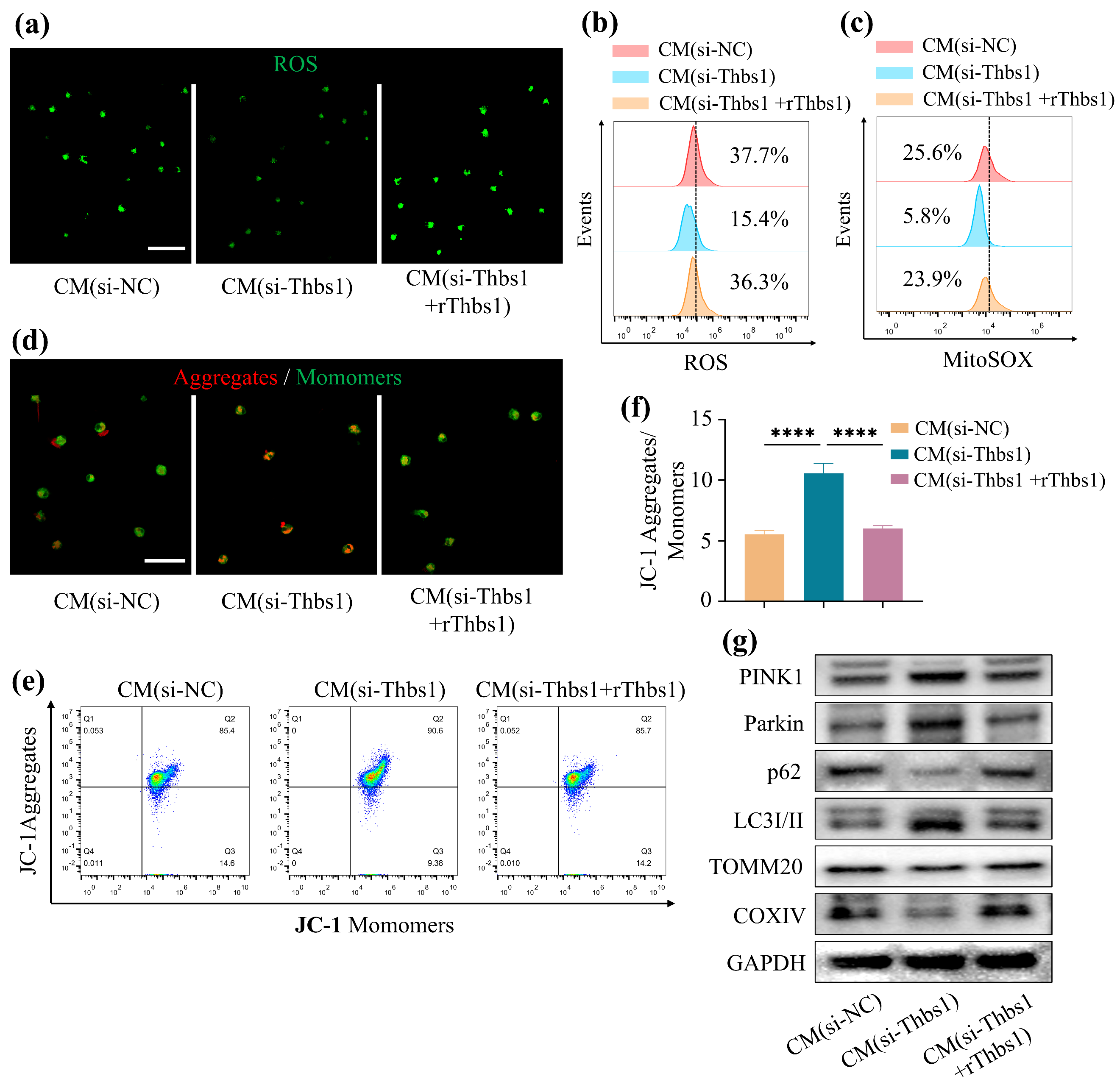


**FIGURE S7. Thbs1 impairs BMDMs' mitochondrial function and mitophagy.**

BMDMs were treated with conditioned medium (CM) from aged BMSCs transfected with si-NC, si-Thbs1, or si‑Thbs1 followed by rThbs1 rescue (si‑Thbs1 + rThbs1). **(a)** Representative IF images of total ROS (n = 3). Scale bar: 50 μm. **(b)** Flow cytometric analysis of total ROS levels (n = 3). **(c)** Flow cytometric analysis of mitochondrial ROS levels (MitoSOX Red) (n = 3). **(d)** Representative IF images of MMP assessed by JC-1 staining (n = 3). Scale bar: 50 μm. **(e, f)** Flow cytometric analysis and quantification of the JC-1 monomer/aggregate ratio (n = 3). Data are presented as mean  ±  SD. Statistical significance (*****p* < 0.0001) was assessed using one-way ANOVA with Šídák’s multiple comparisons test (f). **(g)** Western blot analysis of PINK1, p62, Parkin, LC3I/II, TOMM20, and COXIV protein expression in treated BMDMs (n = 3).


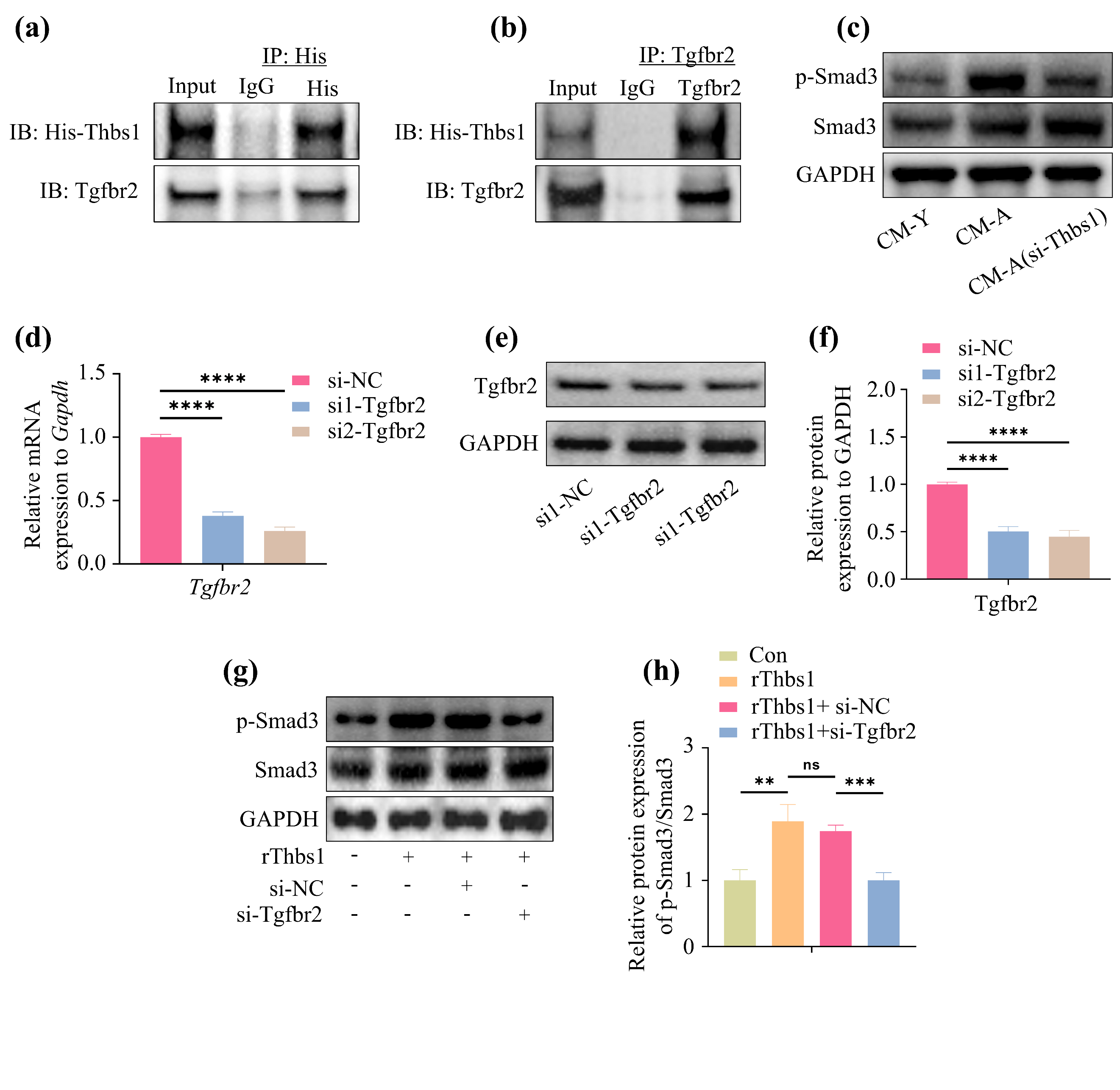


**FIGURE S8. Thbs1 activates TGF-β/Smad3 signaling by binding to the Tgfbr2 on macrophages.**

BMDMs were treated with recombinant Thbs1 (rThbs1) alone or in combination with siRNA-mediated knockdown of *Tgfbr2*. **(a, b)** Immunoprecipitation analysis of the interaction between Thbs1 and Tgfbr2 in BMDMs (n = 3). **(c)** Western blot analysis of p‑Smad3 and total Smad3 in BMDMs treated with CM(Y) or CM(A), with or without *Thbs1* knockdown (n = 3). **(d)** mRNA expression of *Tgfbr2* following siRNA transfection (n = 3). **(e, f)** Western blot analysis **(e)** and quantification **(f)** of Tgfbr2 protein expression (n = 3). **(g, h)** Western blot analysis **(g)** and quantification **(h)** of phosphorylated Smad3 (p‑Smad3) and total Smad3 in BMDMs treated with rThbs1, with or without *Tgfbr2* knockdown (n = 3). Data are presented as mean  ±  SD. Statistical significance (***p* < 0.01; ****p* < 0.001; *****p* < 0.0001; *ns*, not significant) was assessed using one-way ANOVA with Dunnett's test (d and f) or one-way ANOVA with Šídák’s multiple comparisons test (h).


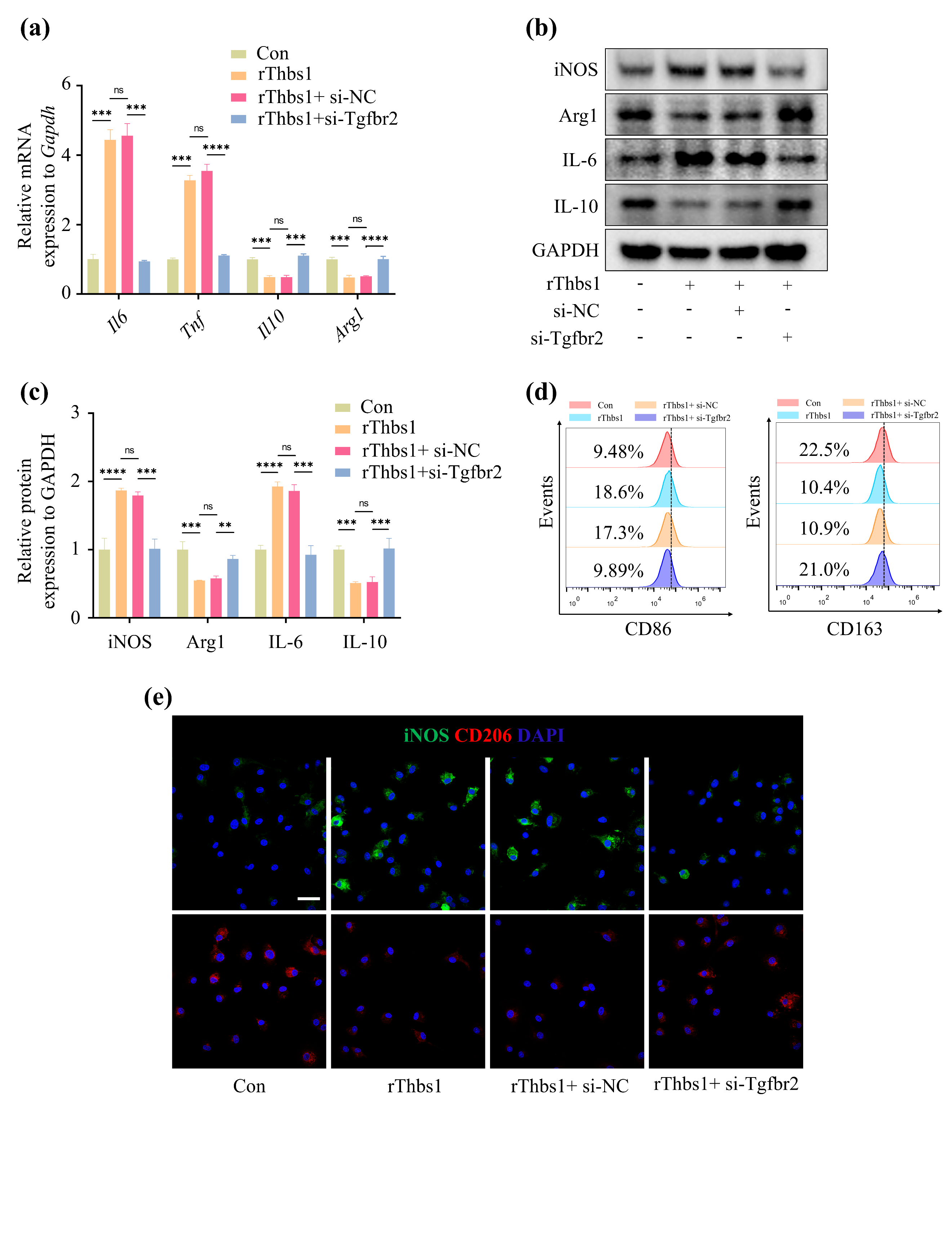


**FIGURE S9. Thbs1 promotes macrophage M1 polarization via TGF-β/Smad3 signaling.**

BMDMs were treated with rThbs1 alone or in combination with siRNA-mediated knockdown of *Tgfbr2*. **(a)** mRNA expression levels of M1-associated (*Il6* and *Tnf*) and M2-associated (*Arg1* and *Il10*) genes in BMDMs (n = 3). **(b, c)** Western blot analysis **(b)** and quantification **(c)** M1 markers (iNOS and IL‑6) and M2 markers (Arg1 and IL‑10) in BMDMs under the indicated conditions (n = 3). **(d)** Flow cytometric analysis of CD86 and CD163 expression (n = 3). **(e)** IF staining of iNOS and CD206 (n = 4). Scale bar: 25 μm. Data are presented as mean  ±  SD. Statistical significance (***p* < 0.01; ****p* < 0.001; *****p* < 0.0001; *ns*, not significant) was assessed using one-way ANOVA with Šídák’s multiple comparisons test (a and c).


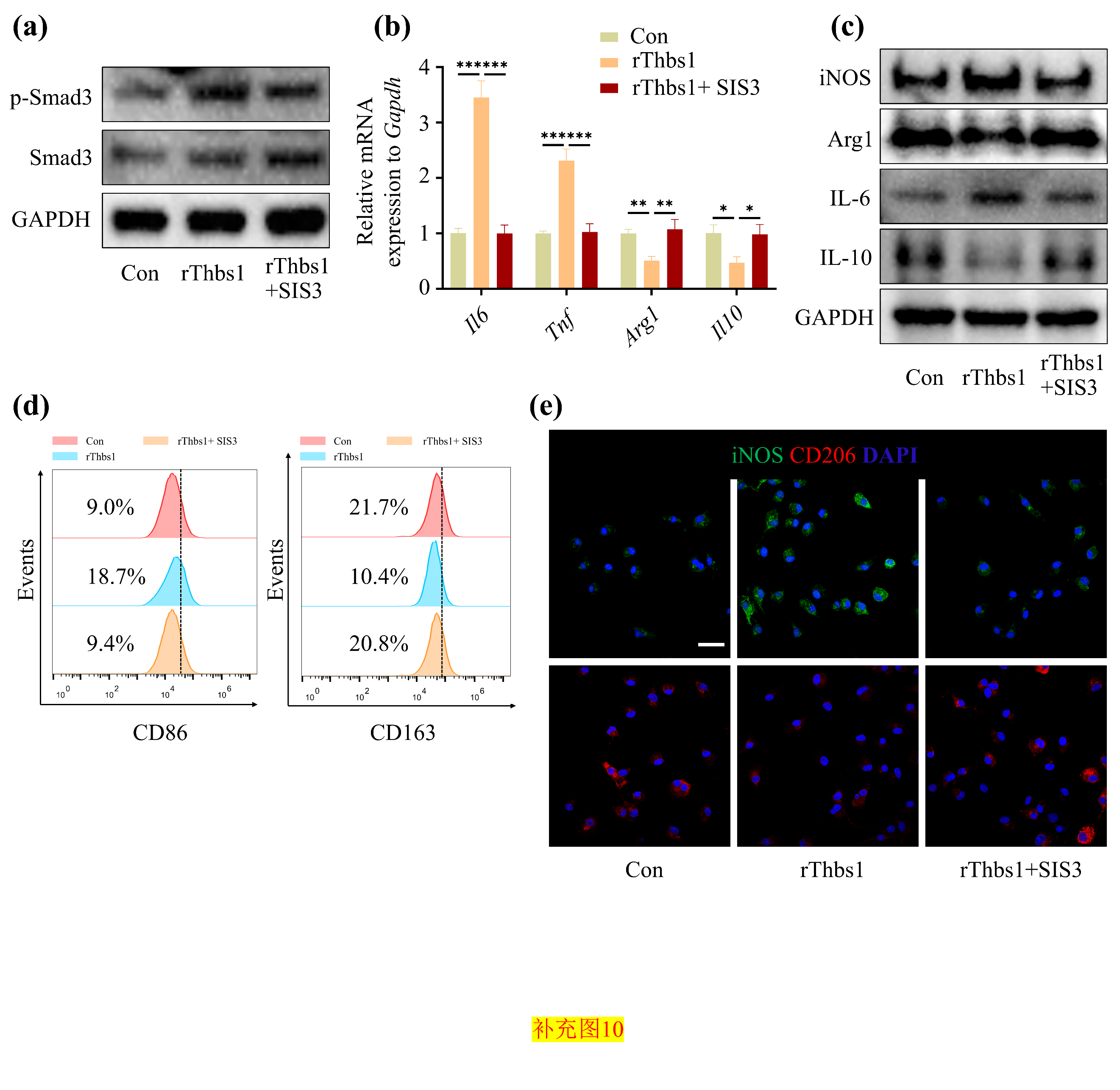


**FIGURE S10. Pharmacological inhibition of Smad3 partially reverses Thbs1‑driven M1 polarization in BMDMs.**

**(a**) Western blot analysis of Smad3 phosphorylation following rThbs1 treatment with or without the Smad3 inhibitor SIS3 (n = 3). **(b)** mRNA expression of M1‑associated (*Il6* and *Tnf*) and M2‑associated (*Il10* and *Arg1*) genes (n = 3). Data are presented as mean  ±  SD. Statistical significance (**p* < 0.05; ***p* < 0.01; ****p* < 0.001) was assessed using one-way ANOVA with Šídák’s multiple comparisons test. **(c)** Western blot analysis of M1 (iNOS, IL-6) and M2 (Arg1, IL-10) markers (n = 3). **(d)** Flow cytometric analysis of CD86 and CD163 expression (n = 3). **(e)** IF images of iNOS (green) and CD206 (red) (n = 4). Scale bar: 25 μm.


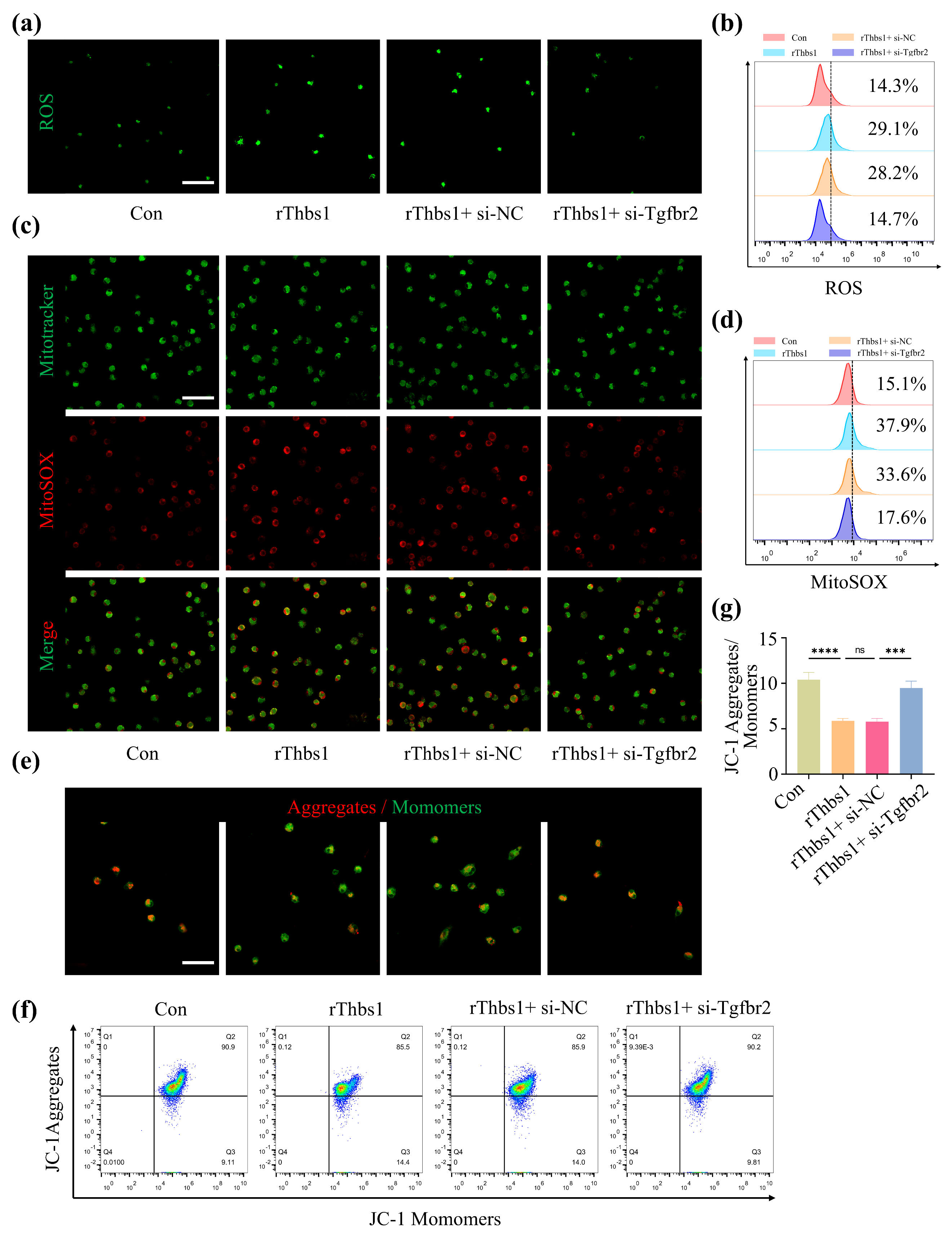


**FIGURE S11. Thbs1 activates TGF-β/Smad3 signaling to disrupt mitochondrial redox balance and MMP in BMDMs.**

BMDMs were treated with rThbs1 in the presence or absence of siRNA‑mediated *Tgfbr2* knockdown.

**(a)** Representative fluorescence images of DCF‑DA staining revealing total ROS in BMDMs (n = 3). Scale bar: 50 μm. **(b)** Flow cytometric analysis of total ROS (n = 3). **(c, d)** Mitochondrial ROS assessment by MitoSOX staining **(c)** and flow cytometry **(d)** (n = 3). Scale bar: 50 μm. **(e)** Mitochondrial membrane potential assessed by JC-1 staining (aggregates, red; monomers, green) in BMDMs (n = 3). Scale bar: 50 μm. **(f)** Flow cytometric analysis of MMP (aggregate fluorescence/monomer ratio) (n = 3). **(g)** Quantification of JC‑1 monomer-to-aggregate ratio in BMDMs (n = 3). Data are presented as mean  ±  SD. Statistical significance (****p* < 0.001; *****p* < 0.0001; *ns*, not significant) was assessed using one-way ANOVA with Šídák’s multiple comparisons test.


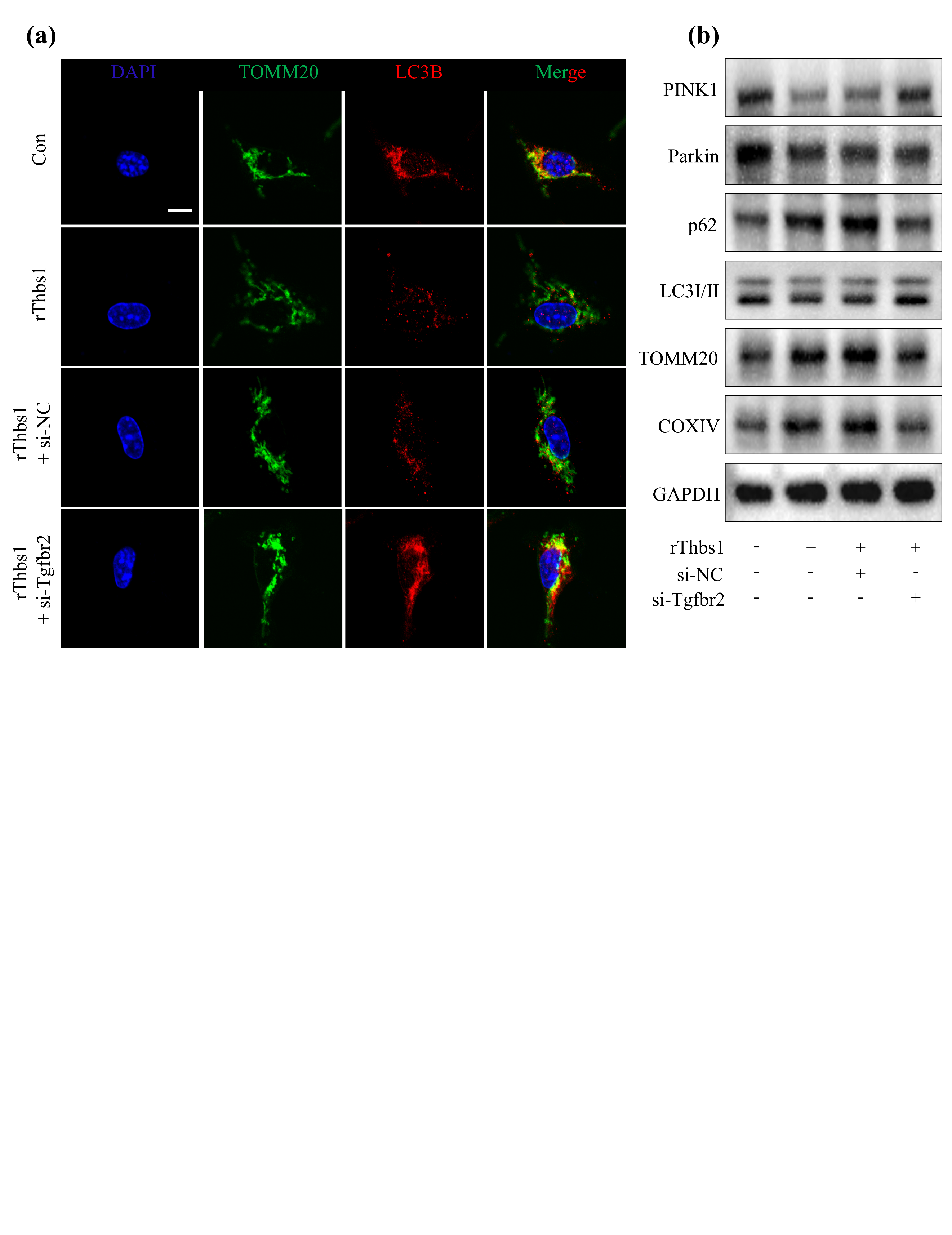


**FIGURE S12. Thbs1 activates TGF-β/Smad3 signaling to suppress mitophagy in BMDMs through TGF‑β/Smad3 signaling.**

BMDMs were treated with rThbs1 in the presence or absence of siRNA‑mediated *Tgfbr2* knockdown. **(a)** Representative fluorescence images of TOMM20 (green) and LC3B (red) in BMDMs (n = 3). Scale bar: 10 μm. **(b)** Western blot analysis of PINK1, Parkin, p62, LC3I/II, TOMM20, and COXIV protein levels in BMDMs (n = 3).


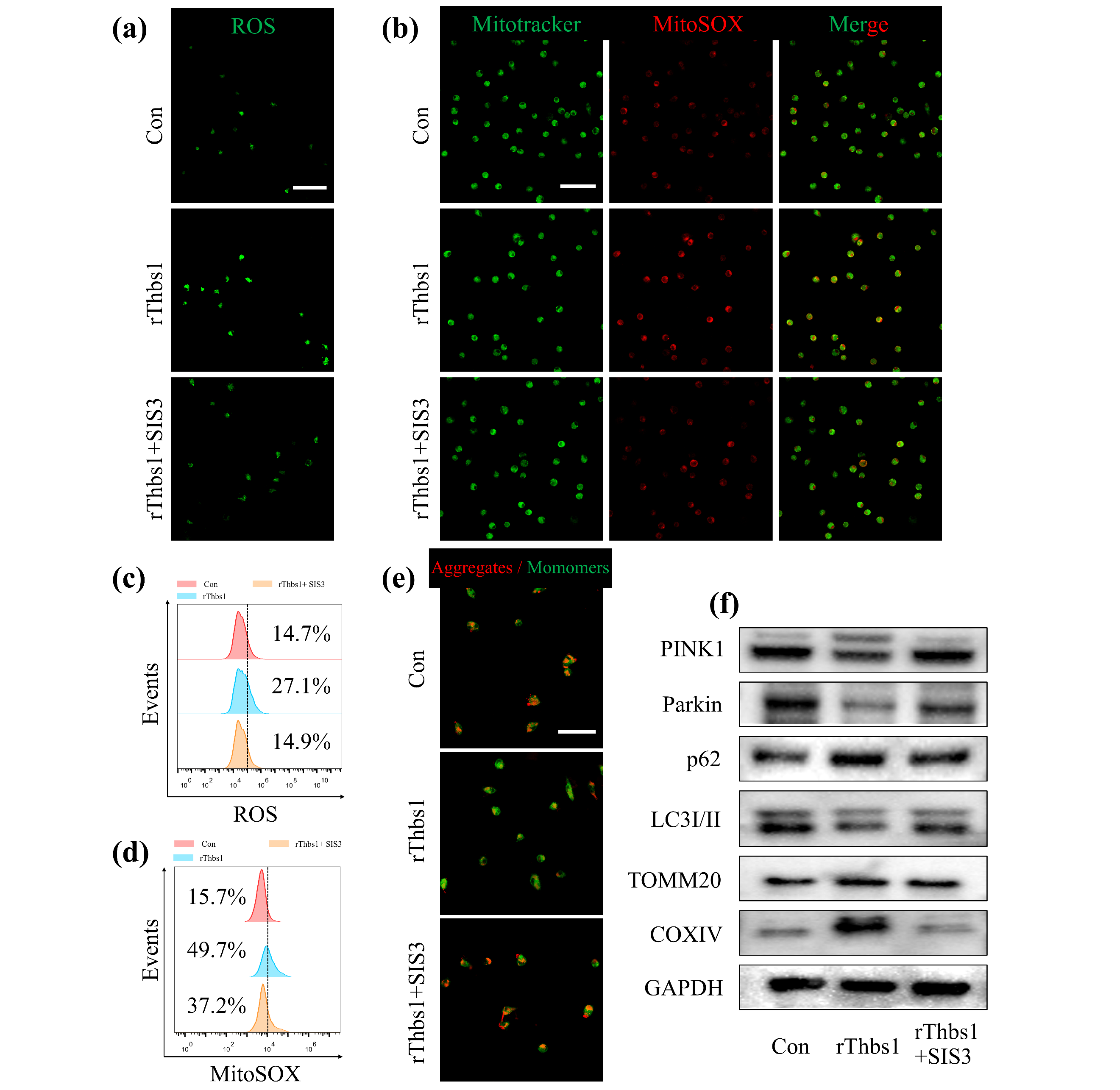


**FIGURE S13. Thbs1 impairs mitochondrial redox balance and mitophagy in BMDMs via Smad3 activation.**

BMDMs were treated with rThbs1 with or without SIS3. **(a)** IF imaging of total ROS (n = 3). Scale bar: 50 μm. **(b)** MitoSOX (red) and MitoTracker (green) staining of mitochondrial ROS (n = 3). Scale bar: 50 μm. **(c)** Flow cytometric analysis of total ROS (n = 3). **(d)** Flow cytometric analysis of mitochondrial ROS (n = 3). **(e)** Representative IF images of MMP assessed by JC-1 staining (n = 3). Scale bar: 50 μm. **(f)** Western blot analysis of mitophagy-related proteins (n = 3).


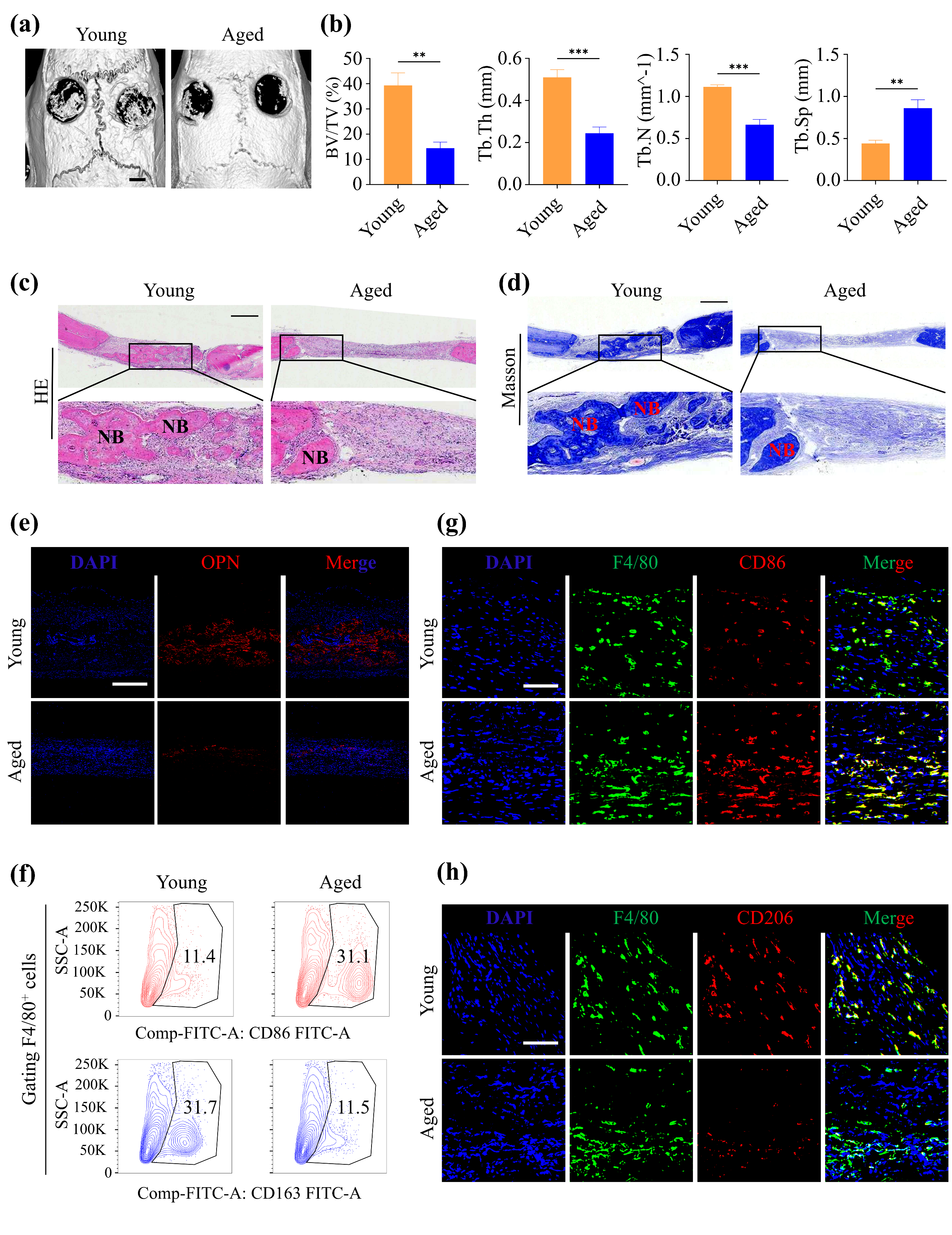


**FIGURE S14. Impaired cranial bone regeneration in aged rats is correlated with enhanced M1 macrophage polarization.**

Young and aged rats were analyzed 1 month after calvarial defect surgery (n = 12 per group). **(a)** Representative micro-CT images of calvarial defects (n = 12). Scale bar: 2 mm. **(b)** Quantitative micro-CT analysis of BV/TV, Tb.Th, Tb.Sp, and Tb.N (n = 6). Data are presented as mean  ±  SD. Statistical significance (***p* < 0.01; ****p* < 0.001) was assessed using unpaired two-tailed Student's *t*-test. **(c, d)** H&E staining **(c)** and Masson's trichrome staining **(d)** of defect regions (NB, new bone) (n = 6). Scale bar: 50 μm. **(e)** IF staining of OPN (red) with DAPI (blue) (n = 6). Scale bar: 25 μm. **(f)** Flow cytometric identification of M1 (F4/80⁺CD86⁺) and M2 (F4/80⁺CD163⁺) macrophages (n = 5). **(g, h)** IF staining of M1 (F4/80⁺CD86⁺) and M2 (F4/80⁺CD206⁺) macrophages in defect sections (n = 6). Scale bar: 25 μm.


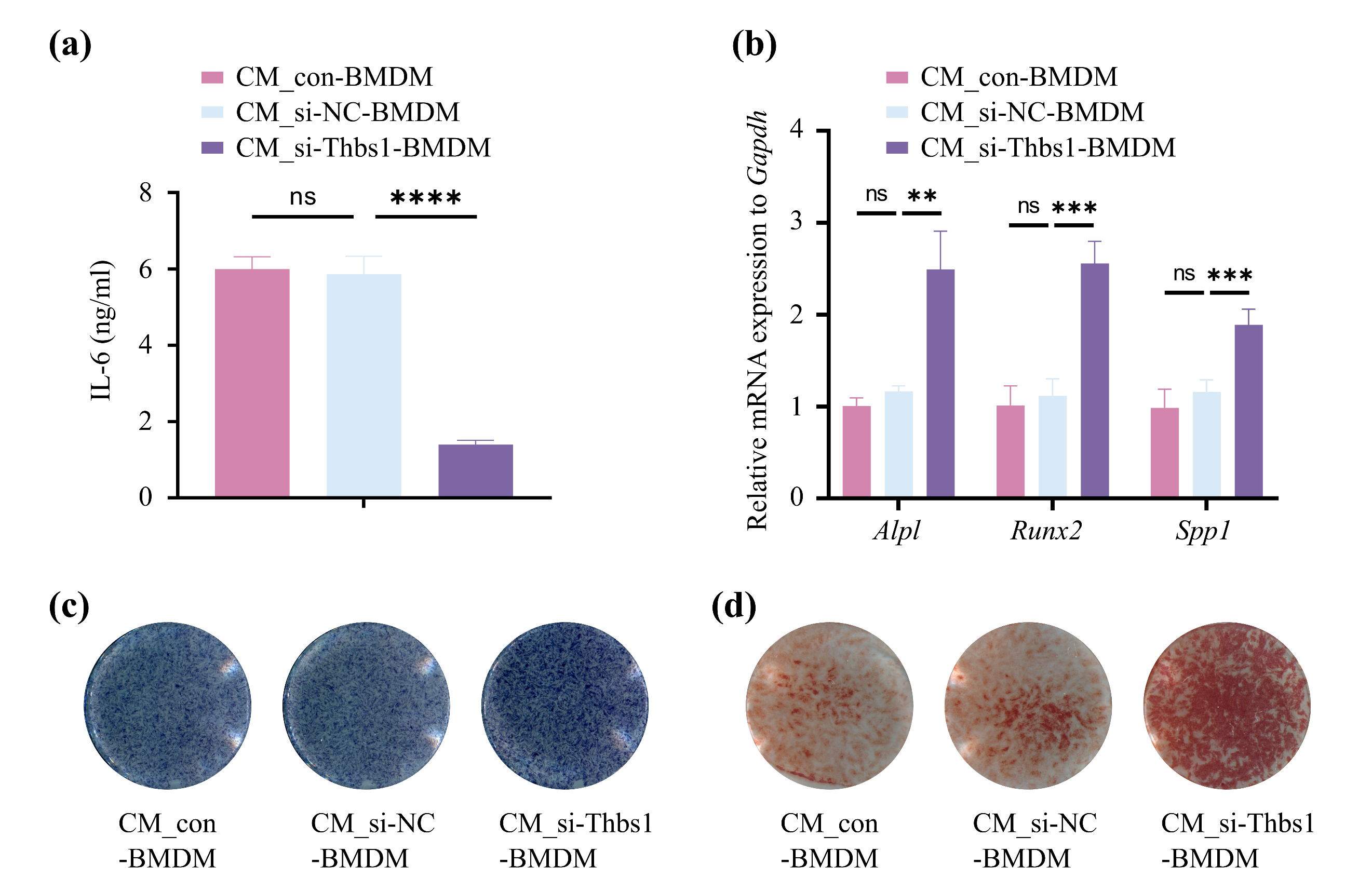


**FIGURE S15. Thbs1 secreted by aged BMSCs suppresses osteogenesis through an M1 macrophage‑mediated feedback loop.**

CM from differently treated aged BMSCs was applied to BMDMs, and the resulting BMDM-CM was used to culture BMSCs. Groups were defined by the initial BMSC treatment: control (CM_con-BMDM), non‑targeting siRNA (CM_si-NC-BMDM), or Thbs1‑targeting siRNA (CM_si-Thbs1-BMDM). **(a)** ELISA analysis of IL-6 secretion in BMDMs (n = 3). **(b)** mRNA expression of osteogenic genes (*Alpl*, *Runx2*, and *Spp1*) (n = 3). **(c)** ALP staining of BMSCs (n = 3). **(d)** ARS staining of mineralized matrix (n = 3). Data are presented as mean  ±  SD. Statistical significance (***p* < 0.01; ****p* < 0.001; *****p* < 0.0001; *ns*, not significant) was assessed using one-way ANOVA with Šídák’s multiple comparisons test (a and b).


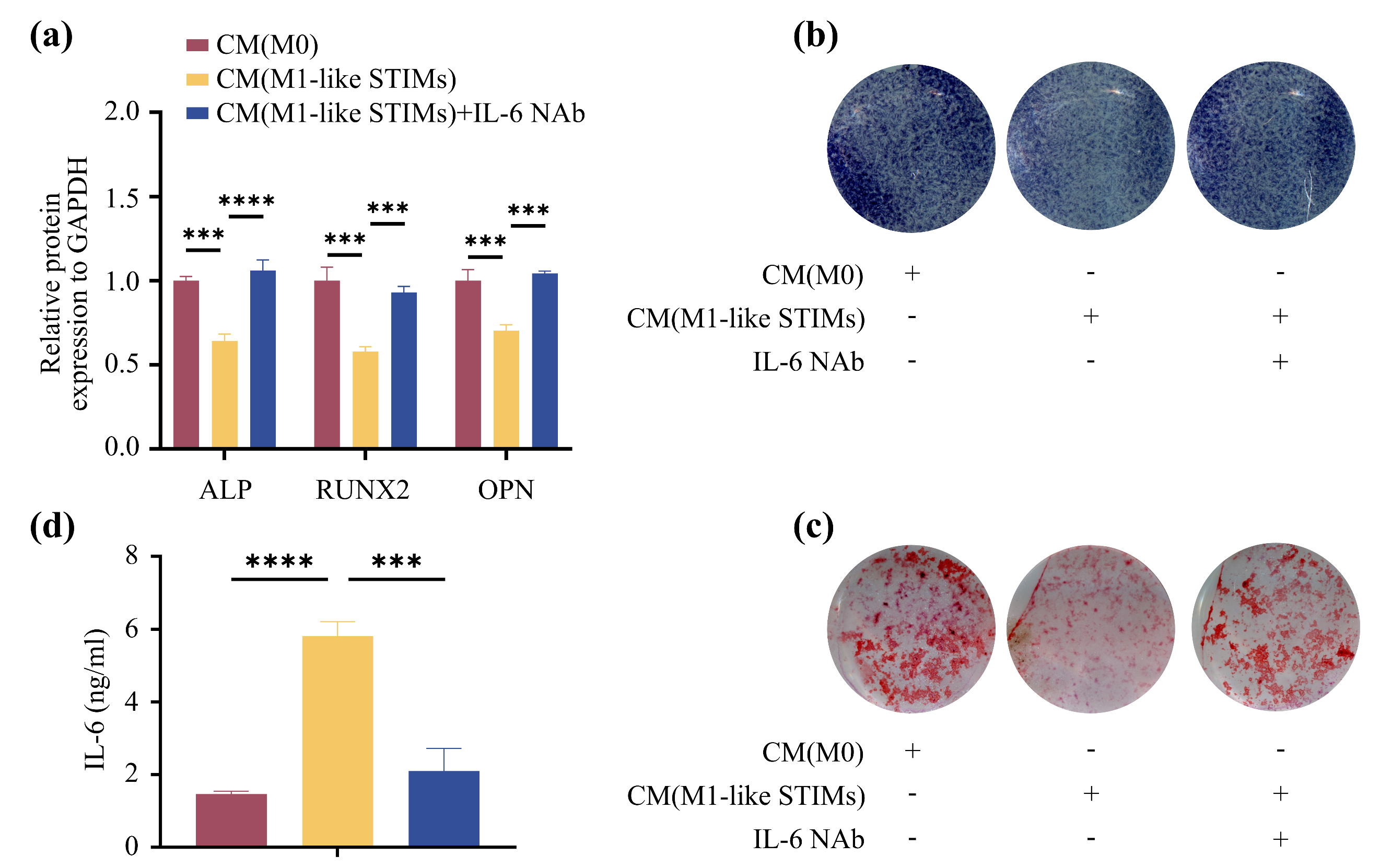


**FIGURE S16. IL‑6 neutralization rescues M1‑like‑STIM‑mediated inhibition of osteogenesis.**

BMSCs were treated with CM from M0 macrophages CM(M0), M1‑like STIMs CM(M1-like STIMs), or M1‑like STIMs supplemented with an IL-6 NAb. **(a)** Quantification of ALP, RUNX2, and OPN protein levels (n = 3). **(b)** ALP and **(c)** ARS staining images (n = 3). **(d)** ELISA analysis of IL-6 secretion in BMDMs (n = 3). Data are presented as mean  ±  SD. Statistical significance (****p* < 0.001; *****p* < 0.0001) was assessed using one-way ANOVA with Šídák’s multiple comparisons test with (a and d).


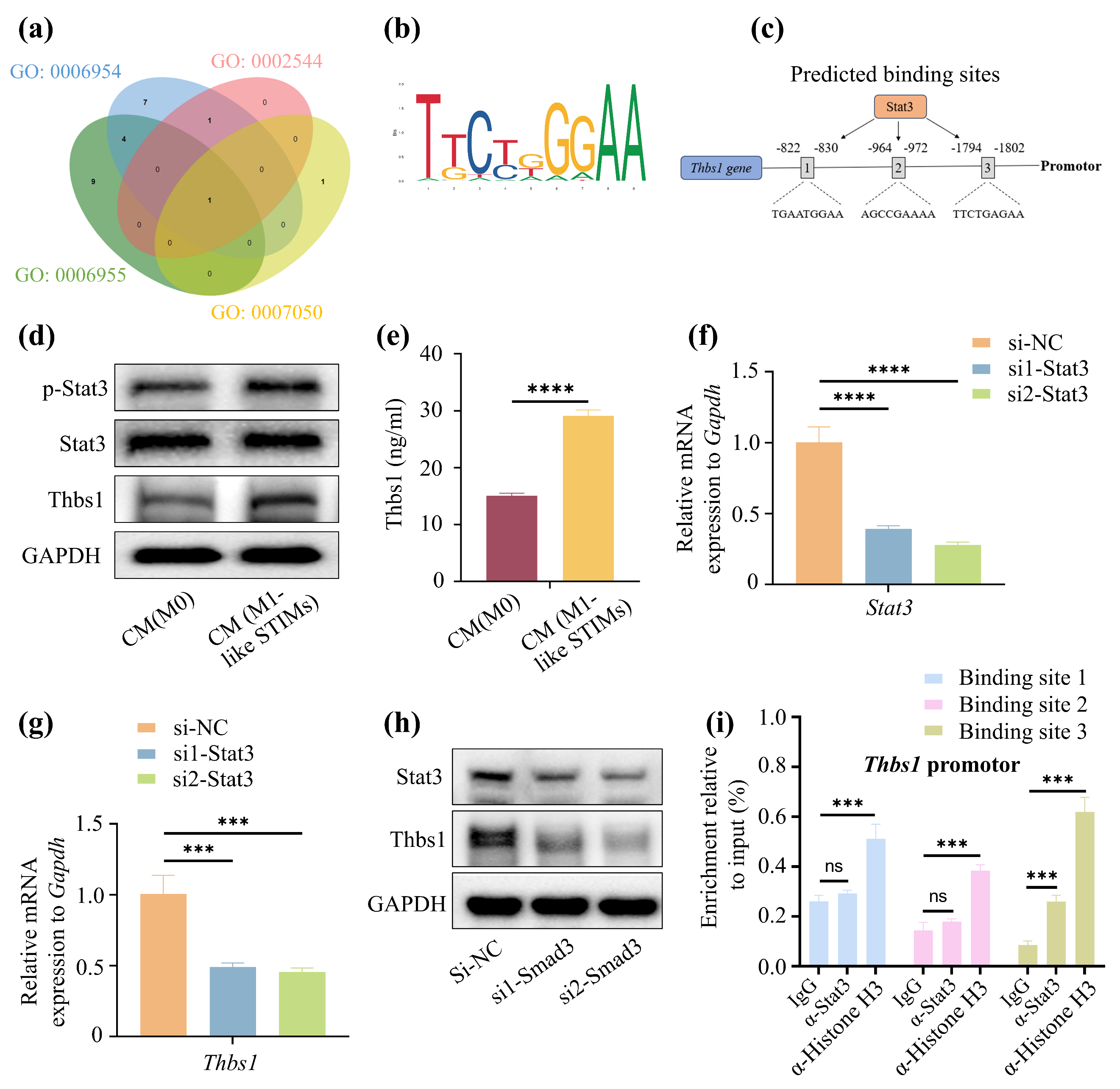


**FIGURE S17. Stat3 transcriptionally activates *Thbs1* to sustain a pro‑inflammatory feedback loop in aged BMSCs.**

**(a)** Venn diagram illustrating overlap of genes associated with key GO terms (immune response, inflammatory response, chronic inflammatory response, and regulation of cell cycle) (n = 4). **(b)** Predicted Stat3 binding motif in the *Thbs1* promoter. **(c)** Schematic representation of predicted Stat3 binding sites. **(d)** Western blot analysis of p-Stat3 and Thbs1 expression (n = 3). **(e)** ELISA quantification of Thbs1 secretion (n = 3). **(f–h)** mRNA and protein analyses following *Stat3* knockdown (n = 3). **(i)** ChIP-PCR analysis of Stat3 occupancy at the *Thbs1* promoter (n = 3). Data are presented as the mean ± SD. Statistical significance (****p* < 0.001; *****p* < 0.0001; *ns*, not significant) was assessed using unpaired two-tailed Student's *t*-test (e) or one-way ANOVA with Dunnett's test (f, g, and i).


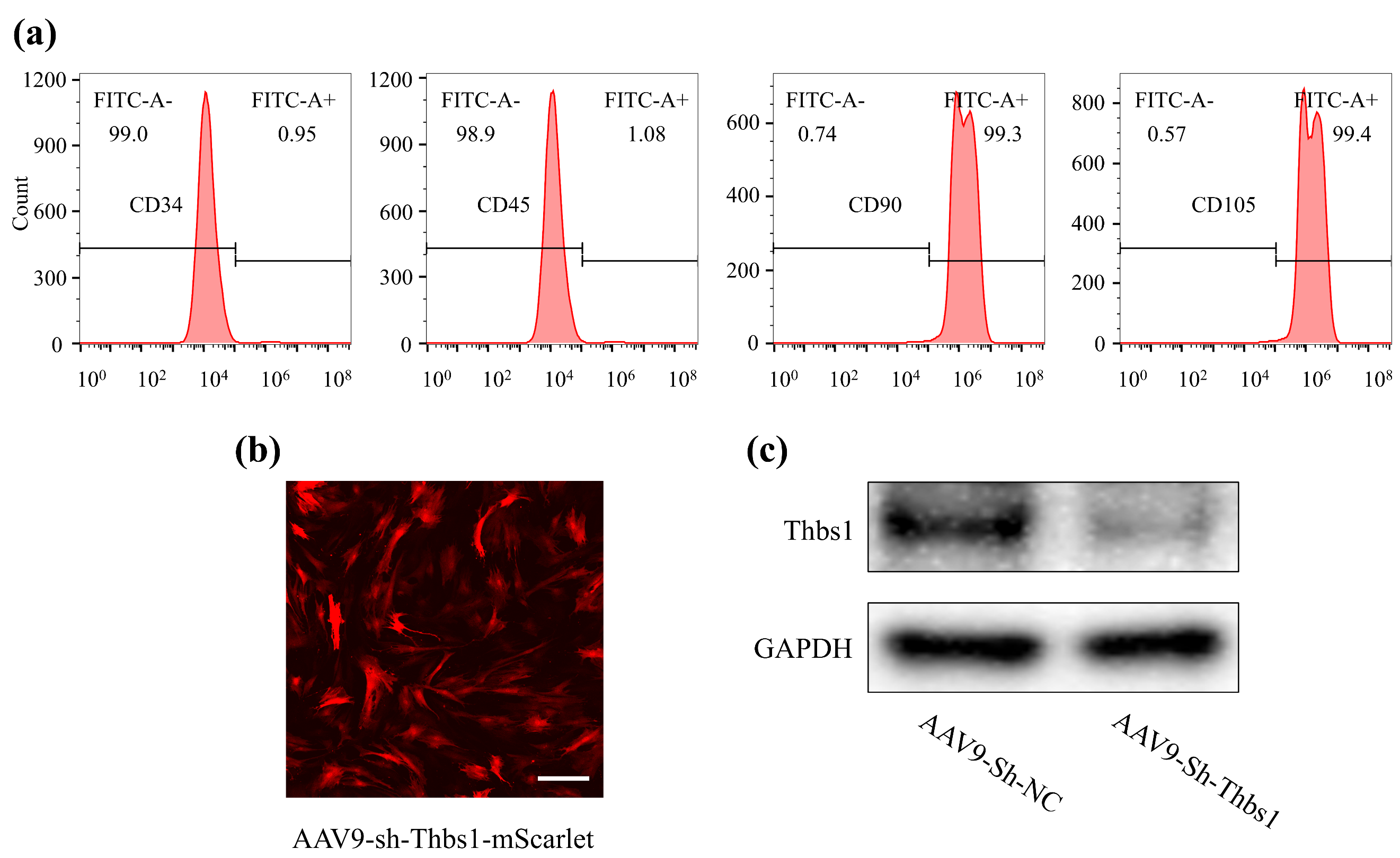


**FIGURE S18. AAV9‑sh‑Thbs1‑mScarlet efficiently silences Thbs1 in BMSCs in vivo.**

**(a)** Flow cytometric analysis of surface markers (CD34, CD45, CD90, and CD105) on BMSCs isolated from the calvarial defect region (n = 5). **(b)** Fluorescence image of mScarlet expression in transduced BMSCs (n = 5). Scale bar: 25 μm. **(c)** Western blot analysis confirming reduced Thbs1 protein expression (n = 5).


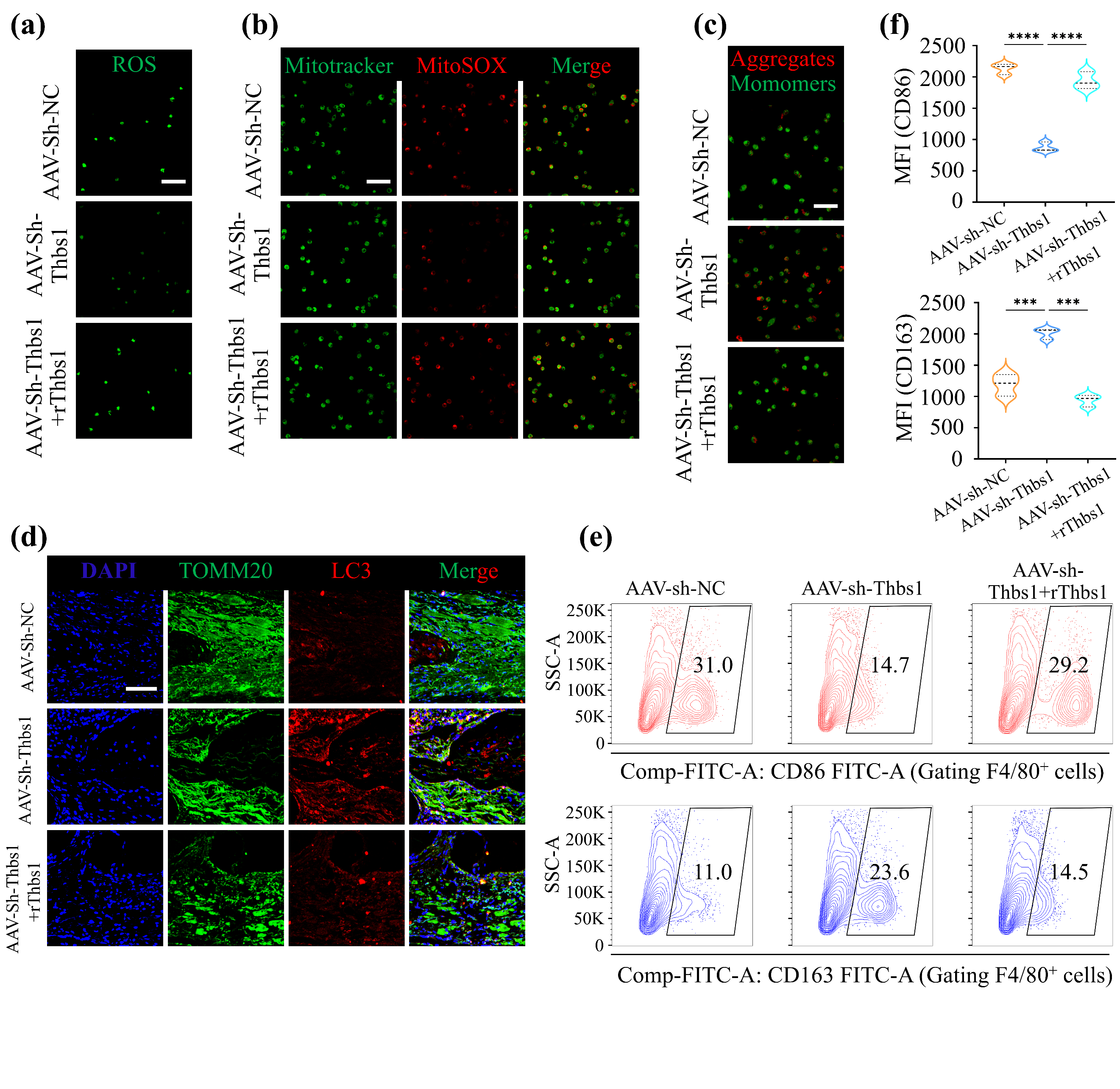


**FIGURE S19. AAV9‑mediated knockdown of BMSC‑derived Thbs1 restores macrophage mitochondrial function, mitophagy, and polarization in aged rat calvarial defects.**

Naturally aged rats were sacrificed 1 month after calvarial defect surgery for analysis (n = 12 per group). **(a)** Representative images of total ROS in macrophages isolated from calvarial defects after AAV9‑mediated Thbs1 knockdown and rThbs1 rescue (n = 5). Scale bar: 50 μm. **(b)** Representative MitoSOX staining image of macrophages from calvarial defects across treatment groups (n = 5). Scale bar: 50 μm. **(c)** Representative images of MMP in defect-associated macrophages (n = 5). Scale bar: 50 μm. **(d)** Colocalization analysis of TOMM20 (red) and LC3B (green) in calvarial defect sections (n = 6). Scale bar: 25 μm. **(e)** Flow cytometric analysis of M1 (CD86⁺) and M2 (CD206⁺) phenotypes in F4/80⁺ macrophages (n = 3). **(f)** Quantification of mean fluorescence intensity (MFI) for CD86⁺ and CD206⁺ in F4/80⁺ macrophages (n = 3). Data are presented as mean  ±  SD. Statistical significance (****p* < 0.001; *****p* < 0.0001) was assessed using one-way ANOVA with Šídák’s multiple comparisons test.
